# Hug1 is an intrinsically disordered protein that inhibits ribonucleotide reductase activity by directly binding Rnr2 subunit

**DOI:** 10.1093/nar/gku1095

**Published:** 2014-11-06

**Authors:** Julie Meurisse, Agathe Bacquin, Nicolas Richet, Jean-Baptiste Charbonnier, Françoise Ochsenbein, Anne Peyroche

**Affiliations:** 1 CEA, iBiTecS, SBIGeM, Gif-sur-Yvette, F-91191, France; 2 CNRS-Université Paris Sud, FRE 3377, Gif-sur-Yvette, F-91191, France; 3 CEA, iBiTecS, SB^2^SM, Laboratoire de Biologie Structurale et Radiobiologie, Gif-sur-Yvette, F-91191, France; 4 CNRS, UMR8221, Gif-sur-Yvette, F-91191, France

## Abstract

Rad53 is a conserved protein kinase with a central role in DNA damage response and nucleotide metabolism. We observed that the expression of a dominant-lethal form of *RAD53* leads to significant expression changes for at least 16 genes, including the *RNR3* and the *HUG1* genes, both of which are involved in the control of nucleotide metabolism. We established by multiple biophysical and biochemical approaches that Hug1 is an intrinsically disordered protein that directly binds to the small RNR subunit Rnr2. We characterized the surface of interaction involved in Hug1 binding to Rnr2, and we thus defined a new binding region to Rnr2. Moreover, we show that Hug1 is deleterious to cell growth in the context of reduced RNR activity. This inhibitory effect of Hug1 on RNR activity depends on the binding of Hug1 to Rnr2. We propose a model in which Hug1 modulates Rnr2–Rnr1 association by binding Rnr2. We show that Hug1 accumulates under various physiological conditions of high RNR induction. Hence, both the regulation and the mode of action of Hug1 are different from those of the small protein inhibitors Dif1 and Sml1, and Hug1 can be considered as a regulator for fine-tuning of RNR activity.

## INTRODUCTION

In response to DNA replication blocks or DNA damage, cells activate coordinated responses collectively referred to as the DNA damage response (DDR), which is mainly orchestrated by the Mec1-Rad53-Dun1 kinase cascade in *S. cerevisiae*. Activation of the ribonucleotide reductase (RNR), which catalyses a rate-limiting step for the *de novo* synthesis of dNTPs (essential elements for DNA synthesis and repair), is part of cellular responses triggered by DDR (1,2). Besides its role in DDR, the essential function of Mec1-Rad53-Dun1 pathway is to regulate RNR to maintain an adequate supply of dNTPs during a normal cell cycle (1,2).

Eukaryotic type Ia RNR consists of two dimeric subunits: the large (R1) catalytic subunit and the small (R2) diferric-tyrosyl radical-generating subunit (3). In budding yeast, R1 is usually an Rnr1 homodimer and R2 is an Rnr2–Rnr4 heterodimer (3). Rnr1–Rnr3 heterodimers also form specifically under DNA damaging conditions (4). The association between R1 and R2 in the cytoplasm is required for RNR activity. This association is dynamic and involves a highly conserved binding site in R2, located at its extreme C-terminus (5,6).

Tightly adjusting the intracellular concentration of dNTPs to meet physiological demands is crucial since unbalanced, elevated or insufficient levels of dNTPs can each lead to a dramatic increase of mutagenesis rates and genomic instability (7–11). RNR is a key enzyme for such regulations, and is itself regulated at different levels. Allosteric regulation of R1 subunit by nucleotides and deoxynucleotides participates in the control of intracellular dNTP levels (12). RNR undergoes further multiple transcriptional and post-transcriptional regulations, in particular at different phases of the cell cycle and in response to DNA damage or replication blocks. The *RNR2*, *RNR3* and *RNR4* genes are under the control of the Crt1 transcriptional repressor, which is itself repressed upon DDR-dependent phosphorylation (13). In contrast, *RNR1* expression depends on the Ixr1 high-mobility group transcription factor (12,14).RNR activity is also regulated by at least two different small protein inhibitors. First, the protein inhibitor Sml1 directly binds cytosolic R1 and inhibits RNR activity (5). This inhibition is released when Sml1 is degraded upon Dun1-dependent phosphorylation (2). Second, Dif1 regulates R2 localization by promoting its nuclear import, which precludes the association of R1 and R2 in the cytoplasm (15,16). Rad53-dependent phosphorylation of Dif1 leads to Dif1 degradation and to the cytoplasmic release of R2 (15,16). In *S. pombe*, the Spd1 protein negatively regulates RNR activity. Spd1 regulates R2 nuclear import but can bind both R1 and R2 (17). *S. pombe* Spd1 shows sequence homology with both Dif1 and Sml1 proteins (15). Synteny analysis suggests that an ancestral locus underwent duplication in *S. cerevisiae* and that the two copies diverged to give rise to *DIF1* on chromosome XII and to *SML1* and *HUG1* genes on chromosome XIII (Supplemental Figure S1). Although *SML1* and *HUG1* are in close proximity with the same orientation, they are regulated independently (18).

Sml1 and Dif1 proteins share a domain, the Sml domain, which is involved in their phosphorylation-dependent degradation (15,16). Dif1 also shares a region of sequence similarity with the first half of the suggested Sml1 RNR1-binding domain (19) but has not been shown to be able to bind R1. By contrast, the Spd1 protein, which binds R1 in *S. pombe*, contains a region of restricted sequence similarity with the last half of the suggested Sml1 RNR1-binding domain, which is very likely responsible for R1 binding and inhibition (17,19). These observations suggest that the specific functions of these weakly related motifs have to be carefully experimentally tackled for each protein. In the same line, although Spd1 regulates R2 nuclear import as Dif1 does, the major restraint of RNR activity *in vivo* by Spd1 could be unrelated to R2 subcellular localization (17).

Hug1, Dif1 and Spd1 share a sequence motif, the Hug domain, which is absent from Sml1 (Supplemental Figure S1). The Hug domain is involved in Dif1 and Spd1 binding to R2 (15,17). Hug1 function is not well defined but its transcript has been shown to be highly induced upon DNA damage in a Rad53-dependent manner (18,20). Deletion of *HUG1* has been reported to rescue the lethality of *mec1* and *rad53* mutants like the deletion of *SML1* or *DIF1* (15–16,18,21). Hence, Hug1 shares phenotypic characteristics with the RNR inhibitors Sml1 and Dif1 (18,22). However, by contrast with Sml1 and Dif1, Hug1 is up-regulated upon DNA damage (18,20). Moreover, the mechanism by which Hug1 could regulate RNR is unknown (22).

Here, we characterized the structural features of Hug1 by nuclear magnetic resonance (NMR) and circular dichroism (CD) spectroscopy and established that it is an intrinsically disordered protein (IDP). We also found that Hug1 directly binds to the small RNR subunit Rnr2 and characterized the surface of interaction. Moreover, we showed that the presence of Hug1 could be deleterious to cell growth under conditions of reduced RNR activity, which indicates an inhibitory effect of Hug1 on RNR activity. This inhibitory effect depends on the residues of Hug1 that are involved in the interaction with Rnr2. However, we showed that, by contrast to the inhibitors Dif1 and Sml1, Hug1 accumulates upon DNA damage or replication blocks or during S phase, which are physiological conditions of high RNR induction. We propose that Hug1 modulates Rnr2–Rnr1 association by binding Rnr2. Hence, being induced similarly to RNR subunits but acting as an inhibitor of RNR, Hug1 could be considered as a rheostat for RNR activity.

## MATERIALS AND METHODS

### Yeast techniques

Standard yeast genetic techniques and media were used (23). To determine growth under various conditions, yeast strains were grown to OD_600_ = 0.1–0.5 before being plated at 10-fold serial dilutions on yeast extract peptone dextrose (YPD) medium with or without drugs. 3-aminotriazol (3AT) was purchased from Sigma.

### DNA and RNA manipulation

The methods for DNA and RNA engineering were essentially those described in (24). Sequences and details of constructions are available upon request.

### Protein sample preparation

Recombinant (His)_6_-GST-TEV site-Hug1 was produced from the pGSTbis-Hug1 plasmid as described for Hsm3 in (25). *Escherichia coli* strain BL21 (DE3) gold containing pGSTbis-Hug1 was grown overnight in 500 ml of Magic Media (Invitrogen). Cells were then harvested by centrifugation, resuspended in Buffer A (50 mM Tris-HCl pH 8, 500 mM NaCl, 5% glycerol, 1% Triton X-100, Complete antiproteases (from Roche), 0.25 mM DTT) and lysed by sonication. The soluble (His)_6_-tagged GST fusion protein was immobilized on Glutathione-agarose (Sigma), washed once with Buffer A, then with Tris-HCl 50 mM pH 8, and then eluted with Tris-HCl 50 mM pH 8 plus 10 mM of glutathione (Sigma). (His)_6_-tagged TEV protease (1% w/w of protease/fusion protein) was added to the eluted proteins. After an overnight incubation at 4°C, Ni-NTA agarose resin (Qiagen) was added in order to trap the (His)_6_-tagged TEV protease and the (His)_6_-tagged GST in the presence of Imidazole 10 mM. Flow-through fraction containing Hug1 was collected and dialysed using a 3000 MWCO membrane to remove imidazole. Further purification was achieved by cation exchange chromatography (Ressource S GE Healthcare, binding conditions: Tris 50 mM pH 8, elution with 500 mM NaCl gradient), yielding to 500 μL of 90% purified protein at 0.44 mg/ml (54.9 μM).

For NMR experiments, recombinant ^15^N- and ^15^N-^13^C-labeled samples were produced by growth in ISOGRO ^15^N or ^15^N-^13^C media (Sigma) respectively. Purification was performed as described above. Ni-NTA flow-through was directly used for NMR recording.(His)_6_-Rnr2 and Rnr4 were produced from plasmids (generous gift from A. Chabes) described in (26). BL21(DE3) gold bacteria were grown overnight in LB media. Cells were harvested by centrifugation, resuspended in Buffer A and lysed by sonication. The soluble proteins were immobilized on Ni-NTA agarose (Qiagen) and purified as described in (26). Imidazole was removed by dialysis using a 6–8000 MWCO membrane. A last purification step was done by anion exchange chromatography (Ressource Q GE Healthcare, binding conditions: Tris 50 mM pH 8, elution with 500 mM NaCl gradient). Fractions containing (His)_6_-Rnr2 and Rnr4 were pooled.

### Circular dichroism experiments

Far-UV CD measurements were recorded on a JASCO J-815 spectropolarimeter on Hug1 samples (10 μM) dissolved in H_2_O by using Quartz SUPRASIL® cells (Hellma) with a path length of 1mm. Far-UV CD spectra of Hug1 were recorded at 10°C, 20°C, 30°C, 40°C, 50°C, 60°C, 70°C, 80°C, 90°C and 100°C. All far-UV CD spectra were recorded from 260–190 nm with a scan speed of 50 nm/min and a time response of 1 s. Equivalent spectra of buffer were recorded and substracted from the spectra of the protein. Molar ellipticities per residue were calculated as follows: $[\theta ]_{res} = \frac{{0,1 \times \theta }}{{l \times C \times n}}$, with θ the ellipticity in mdeg, *l* the cell path length in cm, *C* the concentration in *M* and *n* the number of peptide bonds.

### Calculation of Stokes radius based on Size Exclusion Chromatography (SEC) analysis

SEC experiments were performed with purified recombinant Hug1 using a prepacked Superdex™ 75 HR 10/300 column (GE Healthcare) on an ÄKTA purifier liquid-chromatography system (GE Healthcare). Chromatography was carried out at 4°C in Tris-HCl 10 mM pH 7.4, NaCl 150 mM, DTT 1 mM, at a flow of 0.5 ml/min. Protein elution was monitored by measuring absorbance at 274 nm. Apparent molecular masses of protein eluted from the column were deduced from a calibration curve obtained by loading 200 μL of the following standards: vitamin B12 (1355 Da), RNase A (13.7 kDa, 16.4 Å), Protein A (42 kDa), BSA (66 kDa, 35.5 Å) (Sigma), as well as blue dextran (V0).*K*_AV_ was calculated from $K_{{\rm AV}} = \frac{{V_{\rm e} - V_0 }}{{V_{\rm t} - V_0 }}$, where *V*_e_ is the elution volume for a given protein, *V*_t_ the bed volume of the column and *V*_0_ the void volume of the column. According to the calibration curve obtained with standards as described in (27), we calculated the Stokes radius (*R*_s_) for Hug1 (log(*R*_s_) = −1.5132 *K*_AV_ + 1.7012). *R*_s_ was then compared with the theoretical Stokes radii for a native (*R*_sN_) or fully unfolded (*R*_sU_) protein calculated according to (28).

### NMR spectroscopy experiments

Purified Hug1 protein was concentrated to 50–60 μM in a 3 kDa concentrator (Millipore) and exchange into NMR buffer (NaH_2_PO_4_ 20 mM pH 5; NaCl 50 mM for assignment experiments, Tris-d_11_ 10 mM pH 7.5 (Eurisotop) in D_2_O for titration experiments). 0.1 mM EDTA, 0.1 mM DSS, 0.1 mM NaN_3_ and protease inhibitors (Complete) were added to the 500 μL of sample.NMR experiments were carried out on Bruker DRX-600 and DRX-700 spectrometers equipped with triple resonance cryoprobes at 15°C for assignment experiments, and 9°C and 22°C for titration experiments. The sequential backbone resonance assignments were achieved using standard ^1^H-^15^N HSQC, ^15^N-edited NOESY-HSQC, TOCSY-HSQC, HNCA, HBHA(CO)NH, CBCA(CO)NH, HN(CA)CO and HNCO experiments. Proton chemical shifts (in ppm) were referenced relative to internal DSS and ^15^N and ^13^C references were set indirectly relative to DSS using frequency ratios (29). All NMR data were processed using Topspin (Bruker) and analysed using Sparky (T.D. Goddard and D.G. Kneller, University of California, San Francisco). Values for random coil shifts used in the calculation of secondary H**^α^**, C**^α^**, C**^β^**, H^N^ and C' shifts were taken from studies by (29–32).

### Titration experiments/Kd measurement

The titration was done by adding increasing amounts of concentrated (His)_6_-Rnr2-Rnr4 complex to sample of the ^15^N-Hug1. At each Hug1:Rnr2-Rnr4 ratio, a two-dimensional ^1^H-^15^N HSQC spectrum was recorded, and changes in intensities were measured for all resonances. Following four isolated disappearing peaks, the Kd was measured using equation

{\fontsize{8}{}{\fontsize{8}{11}\selectfont\begin{equation*} [H] = \frac{{ - \left( {[R]_0 - [H]_0 + {\rm Kd}} \right) + \sqrt {\left( {[R]_0 - [H]_0 + {\rm Kd}} \right)^2 + 4{\rm Kd}[H]_0 } }}{2}, \end{equation*}}}

where [*H*] corresponds to free Hug1's concentration and was considered as proportional to the peak intensity of the peak before addition of (His)_6_-Rnr2-Rnr4, [*H*]_0_ total Hug1's concentration and [*R*]_0_ Rnr2–Rnr4 complex total concentration. Kd was fitted using a global fit of the four titration curves.

### *In vitro* pull down assay

Recombinant (His)_6_-GST-TEV site-Hug1 was produced from BL21 (DE3) gold bacteria grown overnight at 37°C in Magic Media (Invitrogen). The (His)_6_-Rnr2 and Rnr4 recombinant proteins were coexpressed in BL21 (DE3) gold bacteria grown overnight at 20°C in Magic Media. Cultures were then pooled and cells were harvested by centrifugation. Cell pellets were resuspended in lysis buffer (Tris 50 mM pH 8, 300 mM NaCl, 0.05% Tween 20 plus protease inhibitor (Sigma)). Lysozyme (1 mg/ml) was added. After 1 h at 4°C, cells were sonicated, and centrifuged at 10 000xg for 20 min. Supernatant (soluble fraction) and pellet (insoluble fraction) were analysed by SDS-PAGE and western blot. Glutathione-agarose (Sigma) beads were incubated with the soluble fraction overnight at 4°C. A first wash step was carried out using lysis buffer and a second one using Tris-HCl 50 mM pH 8. Then proteins were eluted with 20 mM GSH (Sigma) in Tris-HCl 50 mM pH 8. Elution fractions were analysed by western blot following SDS-PAGE using anti-His antibodies (Qiagen).

### Quantitative reverse transcriptase PCR

cDNA was synthesized from 1 μg of purified RNA by using Super Script II protocol (Invitrogen). All reactions were performed in triplicate using an ABI Prism 7300 Sequence detection system (Applied Biosystems). Each reaction contains 12.5 μL MESA Green Master mix (Eurogentec), 300 nm of forward and reverse primer, 10 μL of cDNA (1/100 dilution) and H_2_O to a final volume of 25 μL. Reaction conditions were as followed: 1 cycle of 50°C for 2 min, 95°C for 5 min and 40 cycles of 95°C for 15 s, 60°C for 1 min. *HUG1* mRNA level was compared to *ACT1* mRNA level using ΔΔCt calculations.

### Cell-cycle studies

*bar1Δ* cells were grown to 5.10^6^ cells/ml in YPD. α-factor (1 μg/ml), nocodazole (15 μg/ml) or hydroxyurea (80 or 100 mM) was then added. After 2 h, synchronized cells were harvested, washed three times in fresh YPD and resuspended to no more than 5.10^6^ cells/ml in prewarmed YPD medium. Aliquots corresponding to 10^7^ cells were taken for each time-course point. Cells were fixed using ethanol 70%. After washing with PBS 1X, cells were treated with 1 mg/ml of RNase A (Sigma) for 1 h at 37°C. Cells were then resuspended to at least 5.10^6^ cells/ml in PBS 1X containing 50 μg/mL of propidium iodide (PI) (Sigma) for at least 15 min at room temperature. Harvested cells were then resuspended into PBS 1X containing PI 5 μg/mL and briefly sonicated just before analysis using a FACScalibur flow cytometer (Becton Dickinson). Data were analysed using CellQuest Pro software (Becton Dickinson) and FlowJo (Tree Star).

### Antibodies and immunoblotting detection

The primary antibodies used in this study were the following: 9E10 anti-Myc (generous gift from SPI, CEA Saclay), anti-FLAG monoclonal antibody (Sigma), anti-pentaHis antibody (Qiagen), anti-living colors JL-8 (Clontech), yC-19 anti-Rad53 polyclonal antibody (Santa Cruz), 13D11 anti-V60 ATPase (Molecular Probes), ab9484 anti-GAPDH monoclonal and ab15568 anti-beta Tubulin polyclonal antibodies (Abcam). A polyclonal antiserum was produced against Hug1 using the Hug1 recombinant (see above) protein for immunization of rabbits. HRP-conjugated anti-rabbit or anti-mouse (Promega) were used as secondary antibodies. Detection was performed with ECL chemiluminescent reagents (Amersham). Chemiluminescent signal was quantified using ImageJ (NIH).

### Two hybrid experiments

The procedure was carried out as described in (33). The bait was cloned into the pGBT9 vector and introduced into the Y187 strain. The prey was cloned into the pACT2 vector. Two-hybrid assays were performed as described in (25).

## RESULTS

### 16 genes are differentially expressed in response to Rad53 pathway activation

*RAD53-DL* is a conditional dominant lethal allele of *RAD53* that encodes a hyperactive form of Rad53 and triggers, in the absence of any exogenous genotoxic stress, physiological events normally induced by DNA damage or replication blocks (1–2,34). We reasoned that such a strain provided a unique tool to specifically study Rad53 pathway activation in the absence of the general stress responses triggered by exposure to drugs. Genetic screens using this allele previously led to uncover new regulators of Rad53 pathway (1–2,33,35–36). We monitored global gene expression in a *RAD53-DL* strain using DNA microarray technology. mRNAs were isolated from wild-type and *RAD53-DL* cells grown in YPD for 3 h, converted into cDNA and hybridized to DNA chip arrays after appropriate dye labeling (see Supplemental Materials and Methods for details). Both technical and biological replicates were performed and led us to conclude that expression changes were significant for at least 16 genes (Supplemental Figure S2). We obviously identified *RAD53* and *TRP1*, whose up-regulation is due to the *RAD53-DL* allele construction. It is important to note that among the 14 remaining genes, *RNR3*, *HUG1*, *HSP12*, *YPR015c* and *YNL194c* have been previously found to be transcriptionally induced upon genotoxic insults and/or transcriptionally regulated by the DNA damage response pathways (1–3,18,20,37–40).

### Hug1 is induced upon genotoxic stress

**Figure 1.**
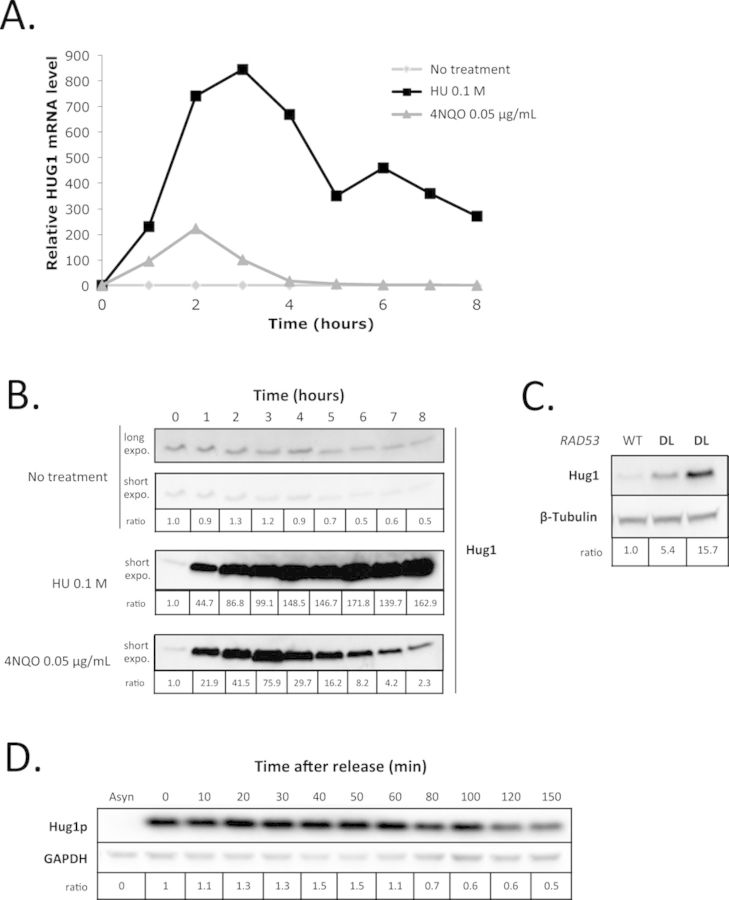
Hug1 level increases in response to DNA damage or replication blocks. (**A**) Exponentially growing cells were either treated with 0.1 M HU (black squares) or 0.05 μg/mL 4NQO (gray triangles) or mock treated (light gray squares). Relative mRNA levels were evaluated using the *ACT1* gene as a standard. Analyses were performed at different time points as indicated. (**B**) Exponentially growing cells were either treated with 0.1 M HU or 0.05 μg/mL 4NQO or mock treated (no treatment). Total protein extracts were prepared at different time points as indicated during genotoxic treatment. Equal amounts of total protein extracts were loaded and analysed by SDS-PAGE followed by western blotting using anti-Hug1 serum. (**C**) Wild-type (WT) or *RAD53-DL* (DL) cells were grown in rich medium. Equal amounts of total protein extracts were loaded and analysed by SDS-PAGE followed by western blotting using polyclonal anti-Hug1 serum and anti-β-tubulin antibodies. (**D**) Exponentially growing wild-type cells were treated with 0.1 M HU for 2 h before release. Total protein extracts were prepared at different time points after release as indicated. Equal amounts of total protein extracts were loaded and analysed by SDS-PAGE followed by western blotting using polyclonal anti-Hug1 serum and monoclonal anti-GAPDH antibodies. Asyn: asynchronous cells.

We next focused on *HUG1*, which exhibits an 8-fold increase in expression in the *RAD53-DL* strain relative to the wild-type (Supplemental Figure S2). We first monitored in a wild-type strain the kinetics of *HUG1* induction upon treatment with the alkylating agent 4-Nitroquinoline 1-oxide (4NQO) or with hydroxyurea (HU) (Figure 1A). We observed that the maximal level of transcripts was reached after 2 h and was higher after 0.1 M HU treatment than after treatment with 0.05 μg/mL 4NQO. We next checked that the level of Hug1 protein was indeed increased in response to DNA damage. It is of importance since it has been recently reported that methyl methanesulfonate-induced transcriptional increases for *RNR1* and *RNR4* does not lead to an increase in soluble proteins due to post-transcriptional regulation (41). Antibodies were raised against Hug1 so that we could monitor its protein level by western blot after HU and 4NQO treatment. We observed that Hug1 level was indeed significantly increased upon genotoxic insults (Figure 1B) in accordance with what was previously reported (3–4,42). Consistently, the level of Hug1 was increased upon *RAD53-DL* induction (Figure 1C). Moreover, following 2 h of HU treatment in wild-type cells, we detected high levels of Hug1 that further increased for 50 to 60 min after release, before slowly decreasing (Figure 1D). Hence, by contrast to what was observed for *RNR1* and *RNR4*, increase of *HUG1* transcripts in response to DNA damage is accompanied by an increase of the level of soluble Hug1 protein.

### Hug1 is regulated during the cell cycle

**Figure 2.**
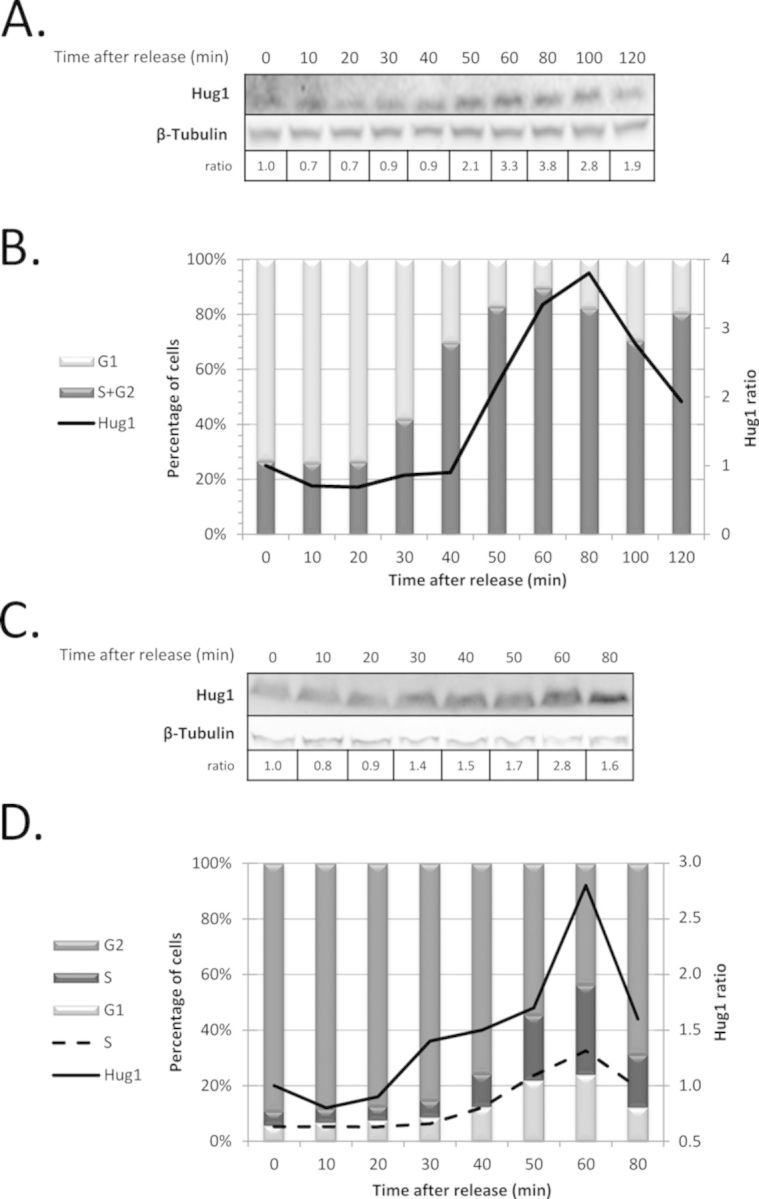
Hug1 level is regulated during the cell cycle. Cells were synchronized during 2 h using alpha-factor (**A**, **B**) or nocodazole (**C**, **D**). At different time points after release as indicated, protein extracts were prepared for western-blotting analysis (A, C) and cell samples were analysed by flow cytometry to quantitate cell-cycle progression (B, D). The chemiluminescent signal obtained in western blot using anti-Hug1 serum and anti-β-tubulin was quantified using ImageJ (NIH). Hug1/β-tubulin signal ratio is indicated below blot images (A, C) and reported as a solid line in quantitative analysis of cell-cycle plots (B, D). Medium gray, dark gray and light grey bars represent the percentages of cells in G2, S and G1 phases respectively, which were calculated from flow cytometry data using FlowJo (Tree Star); the percentage of cells in S phase is also represented by a dashed line (D).

We noticed that the increase in Hug1 level coincides with the induction of RNR activity in response to DNA damage. During this response, cells optimize enzyme activities to promote efficient DNA replication and repair. We reasoned that if Hug1 controls RNR activity, then the level of Hug1, like RNR activity, should be regulated along the cell cycle and during S-phase in particular. To test this prediction, cells were synchronized in G1 or G2/M, and Hug1 level was analysed as a function of time after release. Cell-cycle characteristics were also quantitated by FACS analysis at each time point. Although Hug1 levels were quite low in the absence of genotoxic insult, we observed that Hug1 levels increased during S phase and at the beginning of G2 after release from G1 (Figure 2A and B). Similarly, nocodazole-treated G2/M cells contained low quantities of Hug1, but the levels of Hug1 increased after release from G2/M, especially during the S phase (Figure 2C and D).

Taken together, our observations suggest that the highest levels of Hug1 coincide with a majority of cells being in late S or early G2 phases. These results confirm our prediction that Hug1 level is cell-cycle regulated. Interestingly, the accumulation of Hug1 coincides with physiological conditions of high RNR activity. In order to characterize Hug1's function and protein partners, we investigated its structural properties.

### Hug1 is an intrinsically disordered protein (IDP)

**Figure 3.**
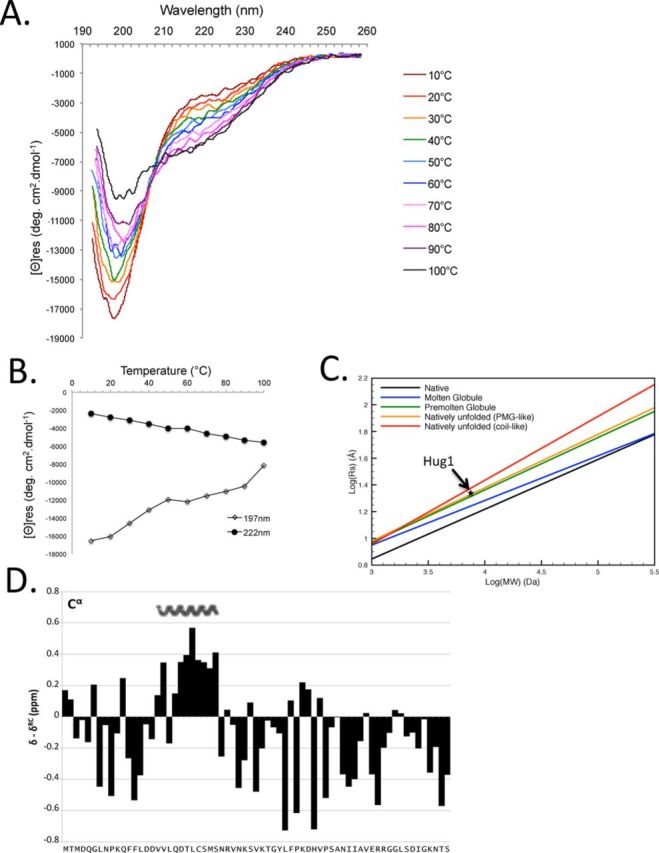
Hug1 is an IDP. (**A**) CD spectra in far-UV region are monitored at several temperatures spanning from 10 to 100°C on purified recombinant Hug1 by using a spectropolarimeter equipped with a water circulation temperature control. (**B**) Effect of temperature on far UV CD spectra of Hug1. Thermal unfolding transition curves are provided by plotting the change in ellipticity at 197 nm and 222 nm from A across temperature. No melting transition is observed for Hug1. (**C**) Hug1's apparent *R*_s_(star) based on its elution time in Supplemental Figure S4 matches that of a natively unfolded protein with a PMG-like structure. *R*_s_, Stokes radius; MW, Molecular Weight. Lines corresponding to five known protein conformations were reported according to (28). (**D**) Chemical shift index calculated for Hug1 ^13^C^α^ nuclei using random coil shift set from (30). Positive values represent α-structure propensity. Nascent alpha-helix is indicated above.

The phylogenic proximity of Hug1 with Spd1, which has been shown to be an IDP, suggested that Hug1 could also be an IDP. Hug1 has several regions of low complexity and is predicted to have limited α-helical and β-strands content (Supplemental Figure S3). Several structural prediction programs (http://www.disprot.org/predictors.php) did not give a high probability of disorder for Hug1 in particular for its central region. However, DisEmBL (Loops/coils prediction) (3,5–6,43) predicted Hug1 to be an IDP (Supplemental Figure S3). We thus experimentally tested this possibility using multiple approaches.IDPs typically have a low content of ordered secondary structure. CD spectroscopy is sensitive to secondary structure. Far-UV CD allows estimations of the α-helical, β-sheet and random coil content of a protein. Figure 3A represents far-UV CD spectra of Hug1 measured at different temperatures. At low temperature, Hug1 is characterized by a far-UV CD spectrum typical of an essentially unfolded polypeptide chain. The spectrum recorded on Hug1 indeed reflects that of a disordered polypeptide with characteristic deep minima in vicinity of 200 nm (minimum at 198nm) and low ellipticity at 220 nm (Figure 3A). Note that the negative ellipticity (−2500°.cm^2^.dmol^−1^) at 222 nm, rather than zero or a positive ellipticity which are taken as indicative of complete disorder, could suggest the presence of some elements of order. Figure 3A shows that the shape and intensity of Hug1 spectrum undergoes considerable changes with temperature, reflecting the disappearance of polyproline II structure, as shown by (44). Figure 3B represents corresponding [θ]_197_ and [θ]_222_ versus temperature dependence. It shows that induced thermal melting did not produce the spectral transition characteristic of cooperative unfolding. The temperature increase is accompanied by the monotonous increase of ellipticity at 222 nm, contrary to the temperature-induced reduction in the content of ordered secondary structure observed in a normal globular protein. In conclusion, CD experiments provide evidence for Hug1 being an IDP.

### The hydrodynamic dimension of Hug1 measured by size-exclusion chromatography suggests a pre-molten globule like structure

We observed that purified recombinant Hug1, which has a calculated 7.5 kDa molecular mass, elutes earlier than expected during size-exclusion chromatography (Supplemental Figure S4). It elutes significantly earlier than 13,7 kDa ribonuclease A, which forms a compact structure. The elution volume of different well-behaved proteins of known molecular weight and Stokes radius (*R*_s_) (5–6,12,45) was used to estimate an *R*_s_ for Hug1 of 21.3 Å based on its elution volume (Supplemental Figure S4 and Materials and Methods), as described in (8–11,13,27). The calculated *R*_s_ of 21.3 Å is unexpectedly large for a protein with a molecular weight of 7500 Da. The large hydrodynamic dimension of Hug1 is consistent with a nonglobular structure of low compactness (12,14,28). A clear relationship between the molecular weight of proteins from five known protein conformations (folded, molten globule, pre-molten globule, natively unfolded ‘pre-molten globule like’, natively unfolded ‘coil-like’) and their predicted Stokes radii has been established (5,13,28). We calculated Hug1's predicted *R*_s_ for each of these classes (Figure 3C) and found the best match with the native unfolded ‘pre-molten globule like’.

### Hug1 has limited secondary structure, with helical propensity in the region spanning V17-S27

We recorded a ^1^H,^15^N heteronuclear single quantum coherence (HSQC) experiment on a uniformly ^15^N-labeled Hug1 sample at pH 5.0. This spectrum showed a narrow distribution of the amide proton chemical shifts as typically observed for IDPs (Supplemental Figure S5A). To further investigate the presence of residual structure, we achieved the sequential backbone resonance assignments using a standard ^15^N-^13^C approach (Supplemental Figure S5A). The chemical shifts obtained were deposited in the BioMagResBank (http://www.bmrb.wisc.edu) under accession number 25044.

Since backbone chemical shifts are very sensitive to the local environment, they can be used as a probe for the presence of secondary structure elements (2,12,31,46). The strategy consists in comparing the experimental chemical shift with a reference value for a random coil conformation for each residue type. Sequence-dependent corrections are applied to take into account slight deviations of the random coil value induced by the preceding and following residues (5,15–16,29–30,32). Chemical shift index obtained for C^α^ nuclei were calculated by using random coil shift set from (30) (Figure 3D). Chemical shift index calculated for H^α^, C^α^, C^β^, H^N^ and C’ by using three different random coil shift sets (29–30,32) are also reported in Supplemental Figure S5B. These indices are consistent with each other and indicate a significant but rather low helical propensity for residues V17-S27 while the rest of the sequence is largely devoid of canonical secondary-structure elements. The presence of helical regions spanning V17-S27 is in agreement with the predictor software (Supplemental Figure S3).

In summary, since Hug1 displays a large hydrodynamic dimension, a lack of secondary structure in far-UV CD, and a collapsed NMR spectrum, Hug1 possesses clear hallmarks of an IDP. We thus established that Hug1 is an IDP, a characteristic shared with Spd1 and Sml1 proteins (17,19,47).

### Hug1 directly binds small RNR subunits

**Figure 4.**
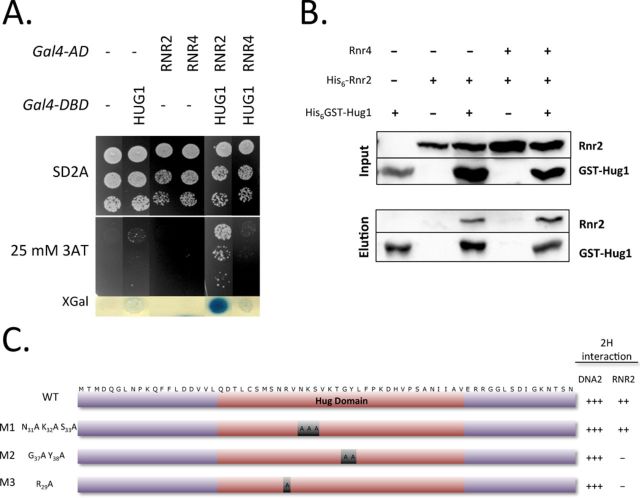
Hug1 directly binds the Rnr2 ribonucleotide reductase subunit. (**A**) Hug1 was fused to Gal4 DNA-binding domain (Gal4-DBD) and Rnr2 or Rnr4 to Gal4 activating domain (Gal4-AD). Empty vectors (−) (pGBT9 and pACT2 respectively) were used as negative controls. Serial dilutions of diploids containing the various combinations of Gal4 fusions were plated in the absence (SD2A) or in the presence of indicated concentrations of 3-Amino-Triazol (3AT) to evaluate transcriptional activation of *HIS3.* Blue color formation in the presence of X-gal indicating transcriptional activation of the second reporter gene *Lac*Z was then monitored. (**B**) Recombinant His-GST-Hug1 (GST-Hug1) was produced in *E. coli* cells. Soluble extracts were loaded onto Glutathion Agarose. Recombinant His_6_-Rnr2 expressed or His_6_-Rnr2−Rnr4 co-expressed was produced in *E. coli* cells. Soluble extracts of *E. coli* cells expressing His_6_-Rnr2 or co-expressing His_6_-Rnr2−Rnr4 were then added. After washing, bound proteins were eluted by adding GSH and analysed by western blotting. Extracts from *E. coli* containing the corresponding empty vectors (-) were used as controls. (**C**) Wild-type (WT) or mutant versions (M1, M2 or M3) of Hug1 were fused to Gal4-DBD. The transformants were mated with Y190 strain containing Gal4-AD-Rnr2 or Gal4-AD-Dna2. Growth of diploids cells was tested in the presence of various concentrations of 3AT to evaluate the *HIS3* reporter activation. Blue color formation in the presence of X-Gal was measured to evaluate transcriptional activation of the *LACZ* reporter gene. Activation of reporter genes was very high (+++), high (++) or undectable (-). Hug domain is indicated in red.

Since Hug1 displayed sequence homologies with both Dif1 and Spd1 (15–17), which were both shown to interact with RNR subunits, we tested for Hug1–RNR interactions. First, we monitored protein interactions using two hybrid assay (2H) as described previously (15–16,25). For this purpose, we introduced *HUG1* and the *RNR* genes into appropriate two-hybrid plasmids (see Materials and Methods). We could detect Rnr2–Rnr2, Rnr4–Rnr4 and Rnr2–Rnr4 interactions using our 2H assay (data not shown) suggesting that Rnr2 and Rnr4 subunits were at least in part functional. We noticed that Rnr4 constructs showed less efficiency than Rnr2 constructs in such experiments. When Hug1 and Rnr2 were used as 2H fusions, we could observe a strong specific activation of the two reporter genes (Figure 4A), suggesting that Rnr2 and Hug1 are effective binding partners. Using Rnr4 as prey and Hug1 as bait, no significant activation signal was observed. To test whether the interaction between Rnr2 and Hug1 was direct, we performed GST-pull down using recombinant proteins. For such assays, soluble extracts from *E. coli* expressing or not His_6_-GST-Hug1 protein were loaded onto Glutathione-agarose beads and then purified recombinant His_6_-Rnr2 protein or purified His_6_-Rnr2-Rnr4 complex was added. Proteins specifically retained onto Glutathione resin were then eluted using GSH. This experiment showed that free Rnr2 and Rnr2 within Rnr2–Rnr4 complex directly bound Hug1 (Figure 4B).

### Characterization of Hug1 binding to R2 subunit

R29, G37 and Y38 in Hug1 are invariable residues within the Hug domain of the Spd1, Dif1 and Hug1 proteins. We tested the ability of the Hug1 R_29_A (M3) and Hug1 G_37_A Y_38_A (M2) mutants to bind Rnr2 in our 2-hybrid assay. We observed that they had both lost Rnr2 binding capacity (Figure 4C and Supplemental Figure S6A). This indicated that these residues are specifically involved in Rnr2 binding since the corresponding mutants kept their ability to bind another 2-hybrid binding partner (Dna2) we had identified in a screen using Hug1 as a bait. Systematic mutagenesis of Spd1 had showed that the *spd1–14* mutant corresponding to R_41_A K_42_A S_43_A within the Hug domain behaves similarly to the *spd1-d* null mutant (17,18). By contrast, the equivalent triple mutant Hug1 N_31_A K_32_A S_33_A (M1) was still able to efficiently bind Rnr2 (Supplemental Figure S6B). Thus, Hug1 binding to Rnr2 *via* its Hug domain likely involves specific molecular determinants, not necessarily shared by Spd1 and Dif1 proteins.

**Figure 5.**
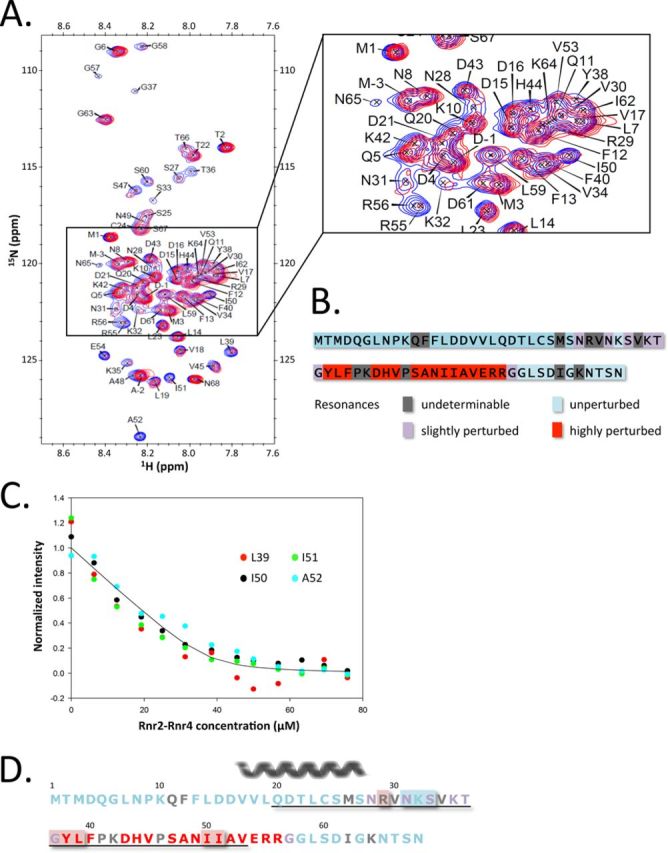
Characterization of the interaction of Hug1 with the Rnr2–Rnr4 complex. (**A**) ^15^N, ^1^H –HSQC spectra of ^15^N-labeled Hug1 at pH 7.5 and 9°C in the absence (blue contours) and in the presence (magenta contours) of saturating amounts of the unlabeled Rnr2–Rnr4 complex (at 1:2 Hug1:Rnr2/4 ratio) are superposed. The concentration of Hug1 is 16.4 μM. (**B**) Schematic representation of Hug1 sequence indicating residues for which resonance in ^15^N, ^1^H –HSQC spectra was highly perturbed (red), slightly perturbed (purple), unperturbed (light blue) upon Rnr2–Rnr4 addition. Residues for which we could not unambiguously determine if resonance was perturbed are indicated in gray. (**C**) Normalized peak intensities measured in ^15^N, ^1^H –HSQC spectra at 22°C were reported as a function of Rnr2–Rnr4 concentration. The concentration of Hug1 is 50 μM. The curve represents the best-fit solution of the exponential equation that describes 1:1 complex formation. The curve corresponds to K_D_ = 1.6 μM ± 0.6 μM. (**D**) Residues of Hug1 involved in Rnr2 binding. Residues that are highly affected by the presence of Rnr2 in NMR experiments are indicated in red, residues that are slightly affected in purple and residues that are unaffected are indicated in light blue. Residues for which mutation abolished 2H interaction with Rnr2 are shown over red background whereas those for which mutation had no effect in our 2H assay are shown over light blue background. Residues for which participation in binding is unknown are indicated in gray. Residues belonging to the Hug domain are underlined. The nascent alpha-helix is indicated above the sequence.

We next carried out ^15^N, ^1^H –HSQC experiments to further characterize the Rnr2–Rnr4 binding to recombinant Hug1. In these experiments Rnr2–Rnr4 binding is indicated by perturbations of the ^15^N, ^1^H –HSQC spectra of the uniformly ^15^N-labeled Hug1 protein. Since Rnr2–Rnr4 proteins were not soluble at acidic pH, these experiments were carried out at pH 7.5. Assignment of free Hug1 resonances under these conditions was achieved thanks to a careful pH titration. Significant variation of peak intensities was observed upon Rnr2–Rnr4 addition showing that Rnr2–Rnr4 indeed binds Hug1 in our experimental conditions (Figure 5A). Saturation was achieved at 1:1.1 Hug1:Rnr2/4 ratio, at 22°C (Figure 5C). At 1:2 Hug1:Rnr2/4 molar ratio and at 9°C (temperature at which the signal to noise ratio was the highest), we observed a strong and significant attenuation of at least 16 peaks (Figure 5A and B). L39, H44, A48, I50, I51, A52, E54, R55 and R56 peak intensities were reduced by more than 70%. The peak intensities of seven other residues were also slightly but significantly reduced, as indicated in Figure 5B. The resonances corresponding to the Hug1 residues bound to Rnr2–Rnr4 are not observed in this spectrum, but this was expected according to the large size of the Rnr2–Rnr4 complex (86 kDa). These experiments clearly showed that (i) Rnr2/4 directly binds recombinant Hug1, (ii) binding involves Y38-F40, D43-V45 and S47-R56 residues in Hug1 and (iii) binding does not involve the first 25 N-terminal amino-acids nor the extreme C-terminal part of Hug1. Interestingly, the binding surface of Hug1 did not include the region found with a residual helical conformation but rather comprised a fully disordered region of Hug1 (Figure 5D).

In order to test whether the Hug1 residues that were identified by NMR as involved in Rnr2–Rnr4 binding could also be identified by two-hybrid assay, we constructed two mutants mutated on the residues for which resonance was highly perturbed after Rnr2–Rnr4 addition: Hug1 Y_38_E L_39_E (M4) and Hug1 I_50_E I_51_E (M5). We observed that they both lost Rnr2 binding capacity (Supplemental Figure S7A and B) suggesting that residues for which resonance is highly perturbed are indeed directly involved in R2 binding.

Titration experiments showed that the Y38-F40 and S47-R56 segments of Hug1 exhibit signal perturbation upon Rnr2/4 addition. Dissociation constants can be obtained by monitoring the signal intensity changes of the backbone amide as a function of the binding partner concentration (15–16,48). Changes in the ^15^N, ^1^H –HSQC spectrum of the uniformly ^15^N-labeled Hug1 upon binding were measured for the I50, I51, A52 and L39 residues at Hug1: Rnr2/4 ratio ranging from 1:0.125 to 1:1.6 at 22°C (Figure 5C). A single K_D_ value of 1.6 ± 0.6 μM was optimized for these residues.

### Hug1 is not required for R2 nuclear localization

Dif1 was clearly shown to be required for the nuclear import of Rnr2–Rnr4 (15,18–19). Since Dif1 and Hug1 both bind the R2 subunit, we investigated a potential role for Hug1 in R2 nuclear import. To localize R2 by immunofluorescence, Rnr2 was epitope-tagged at the chromosomal locus. As expected, Rnr2–3FLAG was mainly detected in the nucleus in G1 phase but relocalized to the cytoplasm upon HU treatment (Supplemental Figure S8). As previously reported, we observed that the nuclear localization of R2 in G1 phase is dependent on the presence of Dif1. By contrast, as earlier reported (15,17,19,22), we could not observe a significant perturbation of the nuclear localization of R2 in the absence of Hug1 (Supplemental Figure S8). Hence, Hug1 is not required for nuclear import and/or nuclear retention of small ribonucleotide reductase subunits.

### Hug1, but not Dif1 is deleterious for some *rnr2* mutants

**Figure 6.**
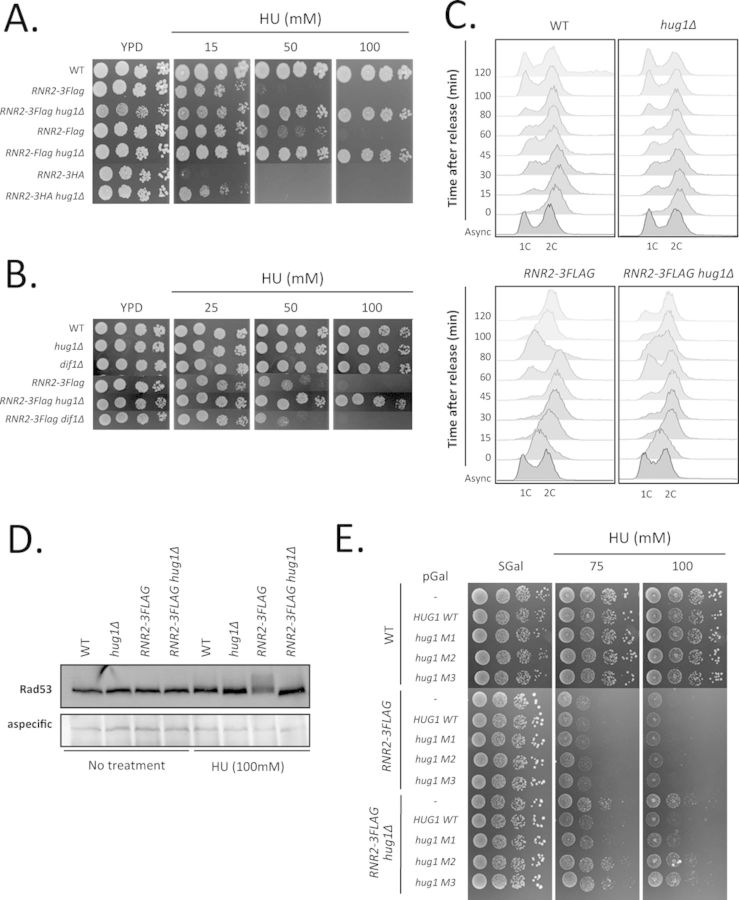
Deletion of *HUG1* specifically alleviates the growth defects of some R2 mutants. (**A**) Invalidation of *HUG1* abrogates the growth defects of epitope-tagged Rnr2 strains in the presence of HU. Five-fold serial dilutions of wild-type (WT) or epitope-tagged Rnr2 strains invalidated or not for *HUG1* were spotted onto YPD plates complemented with hydroxyurea (HU) at various concentrations (15, 50 or 100 mM) and incubated at 30°C for 2 days. (**B**) Invalidation of *HUG1* but not *DIF1* abrogates the growth defects of epitope-tagged Rnr2 strains. Five-fold serial dilutions of exponentially growing cells were spotted onto YPD plates complemented with hydroxyurea (25, 50 or 100 mM) and incubated at 30°C for 2 days. Relevant genotypes are indicated on the left. (**C**) *HUG1* deletion improves the cell-cycle progression of the *RNR2–3FLAG* strain after release from HU block. Wild-type (WT) or *RNR2–3FLAG* strains invalidated or not for *HUG1* were treated for 2 h with HU (80 mM) before release. Cells were fixed with ethanol at indicated time points after release and cell-cycle profiles were analysed by flow cytometry after propidium iodide staining. 1C, 2C: DNA content; 1C corresponds to cells in G1 phase, 2C to cells in G2/M phases. (**D**) Invalidation of *HUG1* decreases the level of Rad53 phosphorylation of the *RNR2–3FLAG* strain after HU treatment. Wild-type (WT) or *RNR2–3FLAG* strains invalidated or not for *HUG1* were treated or not with HU (80 mM) for 2 h. After cell extraction, equal amounts of total protein extracts were loaded and analysed by SDS-PAGE followed by western blotting using polyclonal anti-Rad53 antibodies. (**E**) The suppression of the resistance of *RNR2–3FLAG hug1Δ* cells to HU by reintroducing *HUG1* depends on Rnr2 interaction. Strains whose relevant genotype is indicated on the left were transformed with pGAL-HUG1 WT, mutant (M1, M2 or M3) or the corresponding empty vector. Serial dilutions of transformants were plated on minimal medium containing 2% galactose (SGal) complemented with various concentrations of hydroxyurea (HU) as indicated and grown for 5 days at 30°C.

During the previous experiment, we noticed that the *RNR2–3FLAG* strain was hypersensitive to hydroxyurea (HU) (Figure 6A and B). We next tested the growth of the *RNR2-FLAG* and *RNR2–3HA* strains in the presence of HU. We observed clear growth defects for these strains (Figure 6A). The *RNR2–3HA* strain was the most severely affected showing almost no growth in the presence of 15 mM HU. To further characterize the growth defects of epitope-tagged Rnr2 strains, we investigated their cell-cycle kinetics after release from a 2-h block in HU. We first noted that in exponentially growing cultures, epitope-tagged Rnr2 cells accumulate in the S phase (Figure 6C and Supplemental Figure S9A). After release from HU treatment, the *RNR2–3FLAG* cells progressed more slowly than the wild-type cells; indeed, they enter the subsequent G1 phase at 60 min post-release whereas some wild-type cells are already in G1 at 15 min post-release (Figure 6C). This delay was associated with increased phosphorylation of Rad53 after 2 h of HU treatment, suggesting that the Rad53 pathway is hyperactivated in epitope-tagged Rnr2 strains in response to HU treatment (Figure 6D). Such hypersensitivity suggests that RNR is less active in epitope-tagged Rnr2 strains. Interestingly, it has been established that Rnr2 binds Rnr1 through its C-terminus and the reported structure of Rnr1 complexed with C-terminal nonapeptides of Rnr2 provides a molecular basis for understanding RNR assembly (5–6,17,19). The presence of an epitope at the extreme C-terminus of Rnr2 is likely to perturb the Rnr2–Rnr1 association and thus to reduce cellular RNR activity.

Remarkably, deletion of *HUG1* significantly alleviated the growth defect, allowing almost normal growth of the *RNR2–3FLAG* strain in the presence of 100mM HU (Figure 6A and B). This effect was associated with an improved cell-cycle progression after release from HU block: although exponentially growing *RNR2–3FLAG hug1Δ* cells displayed the same accumulation in the S phase as *RNR2–3FLAG* cells, they entered the subsequent G1 phase 15 min earlier than *RNR2–3FLAG* cells after release from HU block (Figure 6C and Supplemental Figure S9A). Besides, the hyperphosphorylation of Rad53 in the *RNR2–3FLAG* strain was alleviated when *HUG1* was deleted (Figure 6D). These observations suggest that Hug1 negatively regulates RNR activity leading to poor growth in the presence of HU. By contrast, the deletion of *DIF1* did not improve the growth of the *RNR2–3FLAG* strain (Figure 6B). Hence, Hug1 performs a specific function, not shared by Dif1, in regulating RNR activity.

### Hug1's inhibitory effect depends on residues involved in the interaction with Rnr2

In order to characterize the mechanistics of Hug1-mediated inhibition of Rnr2, we expressed wild-type and mutant forms of Hug1 from a plasmid in the *RNR2–3FLAG hug1Δ* strain (Figure 6E and Supplemental Figure S9B). As expected, expressing wild-type Hug1 restored hypersensitivity to HU in the *RNR2–3FLAG hug1Δ* strain (Figure 6E). Expression of the two Hug1 M2 and M3 mutants deficient in Rnr2 binding was not able to restore sensitivity to HU, whereas the other mutant of the Hug domain, Hug1 M1, which is proficient in Rrn2 interaction, restored it. These last results suggest that Hug1 inhibitory effect depends on its interaction with Rnr2. Besides, we observed that Hug1 localization is mainly cytoplasmic (Supplemental Figure S10), which is consistent with the results of (15,17,19,49). Therefore, we propose that Hug1 negatively regulates RNR activity by binding cytoplasmic Rnr2 and thus interfering with Rnr2–Rnr1 association.

## DISCUSSION

### Hug1 is an IDP that could bind multiple partners

Hug1 is a small protein of 68 amino acid residues. The fact that the mutations that prevent Hug1 interaction with Rnr2 are located within the C-terminal part of the Hug domain and that residues located in the N-terminal part of Hug1 are not involved in Rnr2 binding suggests that only a restricted part of the protein is important for Rnr2 binding. The three-dimensional architecture of Hug1 is best defined as that of an IDP, whose significance remains to be determined. NMR studies on Hug1 show that one region of Hug1 exhibits a weak degree of backbone order (V17-S27). However, residues within this region do not participate in R2 binding. By contrast, the long alpha helix within the overall disordered Sml1 protein participates in R1 binding (17–20). It will be of interest to see whether this region of Hug1 is involved in other aspects of Hug1 functions. One attractive hypothesis could be that this region is involved in the association with another partner. Using Hug1 as a bait in a 2-hybrid screening, we have identified a part of Dna2, a multitasking nuclease/helicase protein involved in DNA replication and DSB repair (see (15–17,21,50–52) for reviews). Remarkably, we have shown that Hug1 molecular determinants are different for Rnr2 and Dna2 binding. Interestingly, it has been recently shown that the intrinsically disordered Spd1 protein not only binds both R1 and R2 RNR subunits but also interacts with the processivity factor proliferating cell nuclear antigen (PCNA) complexed onto DNA (53). Due to their intrinsic flexibility, IDPs are often involved in numerous interactions and serve as nodes in protein interaction networks (54). Thus, the IDP nature of Hug1 likely explains how this small protein could be able to bind both the R2 RNR complex and the Dna2 protein.

### Conservation and divergence of the Hug domain in the Spd1, Dif1, Sml1 and Hug1 families of RNR regulators

The *DIF1* and *HUG1/SML1* loci are syntenic and arose from a whole genome duplication event in an ancestor of *S. cerevisiae*. The *A. gossypii* Aer122c protein has been proposed to be close to the ancestor protein (15,18,20). To date, no ortholog of Sml1 or Hug1 has been clearly identified. Syntenic genomic organization suggests that both Dif1-like and Sml1-like proteins exist in almost all post-whole genome duplication (WGD) species (Supplemental Figures S11 and S12A). Searching for Hug1 homologs, we identified proteins of 70 to 78 residues in *Naumovozyma dairenensis*, *Naumovozyma castellii*, *Kazachstania africana* and *Kazachstania naganishii* sharing from 11% to 22% of sequence identity with *S. cerevisiae* Hug1.

Sequence identities within Spd1, Dif1, Sml1 and Hug1 families are weak. High divergence precludes an unambiguous definition of domains or motifs. The Hug domain was defined on sequence similarities (15,18,22). This domain is well conserved in Dif1 and Spd1 orthologs but clearly more divergent in Hug1 orthologs (16,18,20). Aer122c and Dif1 orthologs contain a conserved GMR(I/V)R(K/Q)(S/A) sequence within the Hug domain which has almost disappeared in Hug1 orthologs (Supplemental Figure S12B). Both Aer122c, Dif1-like and Hug1-like proteins comprise a GY motif within the Hug domain. We noticed that immediately downstream of the GY motif the diversity of sequence is high for the Aer122c and Dif1 families, even for close species, whereas GY(L/S)(F/L)PK(D/E) can be highlighted in Hug1 family members (Supplemental Figure S12B). These differences could indicate functional divergence. Our NMR experiments showed that residues at the C-terminal part of the Hug domain, which are not conserved in the Aer122c and Dif1 families, are involved in Hug1 binding to Rnr2. However, residues R_29_, G_37_ and Y_38_ of Hug1, which are present in the Aer122c and Dif1 families, are also involved in the interaction with R2. Thus our results suggest that precise molecular determinants of R2 binding are probably different for different Hug domains. We propose that the Hug domain includes different regions, which are characterized by different conserved residues, namely Hug1 and Dif1 consensus sequences. Consistent with this proposition, it has been recently reported that the Spd1-PCNA interaction involves a somewhat degenerate PIP (PCNA Interaction Protein) that is included within the Hug domain (22,53).

### Hug1 participates in fine-tuning feedback regulation of RNR activity

The precise control of intracellular dNTP pools is required for faithful duplication of the genome and for coping with DNA lesions (10–11,34). In budding yeast, dNTP levels increase by about 3-fold upon entry into S-phase relative to G1 levels and show a 3–5-fold increase in response to DNA damage relative to an unperturbed S phase (7,33,35,36). This is primarily achieved *via* multilayered controls of RNR activity. In yeast, RNR is regulated through allosteric regulation, gene expression, subcellular localization and small inhibitory proteins including Sml1. The R1-R2 association is weak and dynamic. For mouse and *E. coli* RNRs, the dissociation constant for the R1-R2 complex was reported to be around 0.1 μM (55); the interaction between R1 and R2 in the yeast RNR appears much weaker (5). Here, we reported a dissociation constant of 1–2 μM for the Hug1–(Rnr2–Rnr4) complex. Given that the affinity between R2 and R1 is an order of magnitude greater than the affinity between R2 and Hug1, the huge accumulation of Hug1 we observed upon genotoxic insults could significantly modulate R1-R2 association.

Note that roughly comparable RNR-protein inhibitor dissociation constants have been reported even though molecular mechanisms of inhibition of other RNR protein inhibitors are different. Experimental K_D_ value was reported to be 2.4 μM for the Spd1-Cdc22 (R1) complex (56) whereas the Sml1 protein specifically binds to the yeast Rnr1 protein with a dissociation constant of 0.4 μM (5). The dissociation constant for the Dif1-(Rnr2–Rnr4) complex was reported to be 0.6 μM (15).

It is worth mentioning that high RNR activity, and thus high concentration of dNTP, is restricted to S-phase and is transient in response to DNA damage or replication blocks. The continuous presence of high concentrations of dNTP during the cell cycle impairs cell proliferation (11). Limitation of dNTP concentration in G1 phase could be important for normal activation of replication origins and thus normal cell-cycle progression (11). Furthermore, in the presence of constitutively high concentrations of dNTP, the DNA damage checkpoint is inactive (11). It is established that dATP feedback inhibition accounts for coupling dNTP production to their use (12) but RNR activity is clearly further regulated.

**Figure 7.**
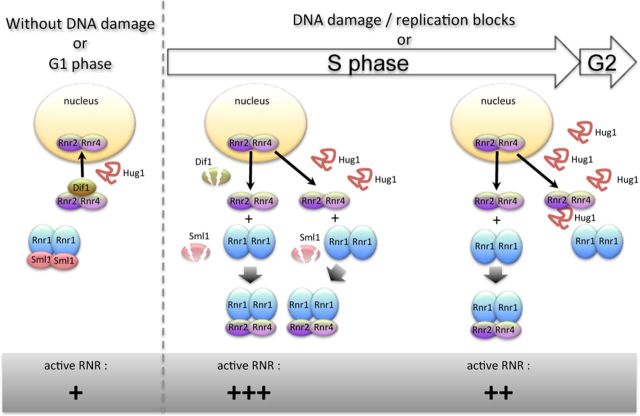
Proposed model of action for Hug1 in RNR regulation. In G1 phase and in the absence of DNA damage, Dif1 facilitates the nuclear import of Rnr2–Rnr4, leading to the accumulation of Rnr2–Rnr4 in the nucleus. Sml1 inhibits RNR activity by binding Rnr1. Upon DNA damage or entry into S phase, the degradation of Dif1 and Sml1 reduces the nuclear import of Rnr2–Rnr4 and induces the release of Rnr1, respectively. R1 and R2 complexes associate in the cytoplasm. Toward the end of S phase or of DNA damage repair, Hug1 accumulates and then binds R2, precluding in part R1-R2 association and thus leading to a decrease of RNR activity. For clarity, Wtm1-dependent nuclear retention of Rnr2–Rnr4 is not indicated.

Hug1 accumulates along S phase and upon DNA damage. Hence, Hug1 accumulates under physiological conditions that lead to high RNR activity. The peak of Hug1 abundance during the cell cycle likely occurs slightly later than the peak of RNR activity. Hug1 is able to directly bind R2 and is likely to inhibit RNR activity by modulating the R1-R2 association and/or the RNR complex architecture as it has been recently proposed for Spd1 (17). Such an effect would be especially strong in epitope-tagged Rnr2 strains in which Rnr2 binding to Rnr1 is already impaired. We indeed observed that Hug1 is deleterious for the growth of such strains and that this inhibitory effect depends on the ability of Hug1 to bind Rnr2. We thus propose that Hug1 participates in the feedback regulation of RNR and helps to turn off RNR activity. We provide a model recapitulating how Hug1 could participate in RNR regulation (Figure 7). This study contributes a further layer to the exquisite multilayer system of RNR regulation.

## SUPPLEMENTARY DATA

Supplementary Data are available at NAR Online.

SUPPLEMENTARY DATA
