# Supplementary material for: Hug1 is an intrinsically disordered protein that inhibits ribonucleotide reductase activity by directly binding Rnr2 subunit
Source: Nucleic Acids Res. 2014 Nov 6;42(21):13174–85. doi: 10.1093/nar/gku1095 (PMC4245953; doi:10.1093/nar/gku1095)
Supplement: SUPPLEMENTARY DATA [file supp_42_21_13174__index.html]

Hug1 is an intrinsically disordered protein that inhibits ribonucleotide reductase activity by directly binding Rnr2 subunit — Hug1 is an intrinsically disordered protein that inhibits ribonucleotide reductase activity by directly binding Rnr2 subunit — SUPPLEMENTARY DATA 

# Hug1 is an intrinsically disordered protein that inhibits ribonucleotide reductase activity by directly binding Rnr2 subunit

## SUPPLEMENTARY DATA

**Files in this Data Supplement:**

- SUPPLEMENTARY DATA
