# Supplementary material for: Hug1 is an intrinsically disordered protein that inhibits ribonucleotide reductase activity by directly binding Rnr2 subunit
Source: Nucleic Acids Res. 2014 Nov 6;42(21):13174–85. doi: 10.1093/nar/gku1095 (PMC4245953; doi:10.1093/nar/gku1095)
Supplement: SUPPLEMENTARY DATA [file supp_gku1095_nar-03636-h-2013-File009.pdf]

Supplemental Data

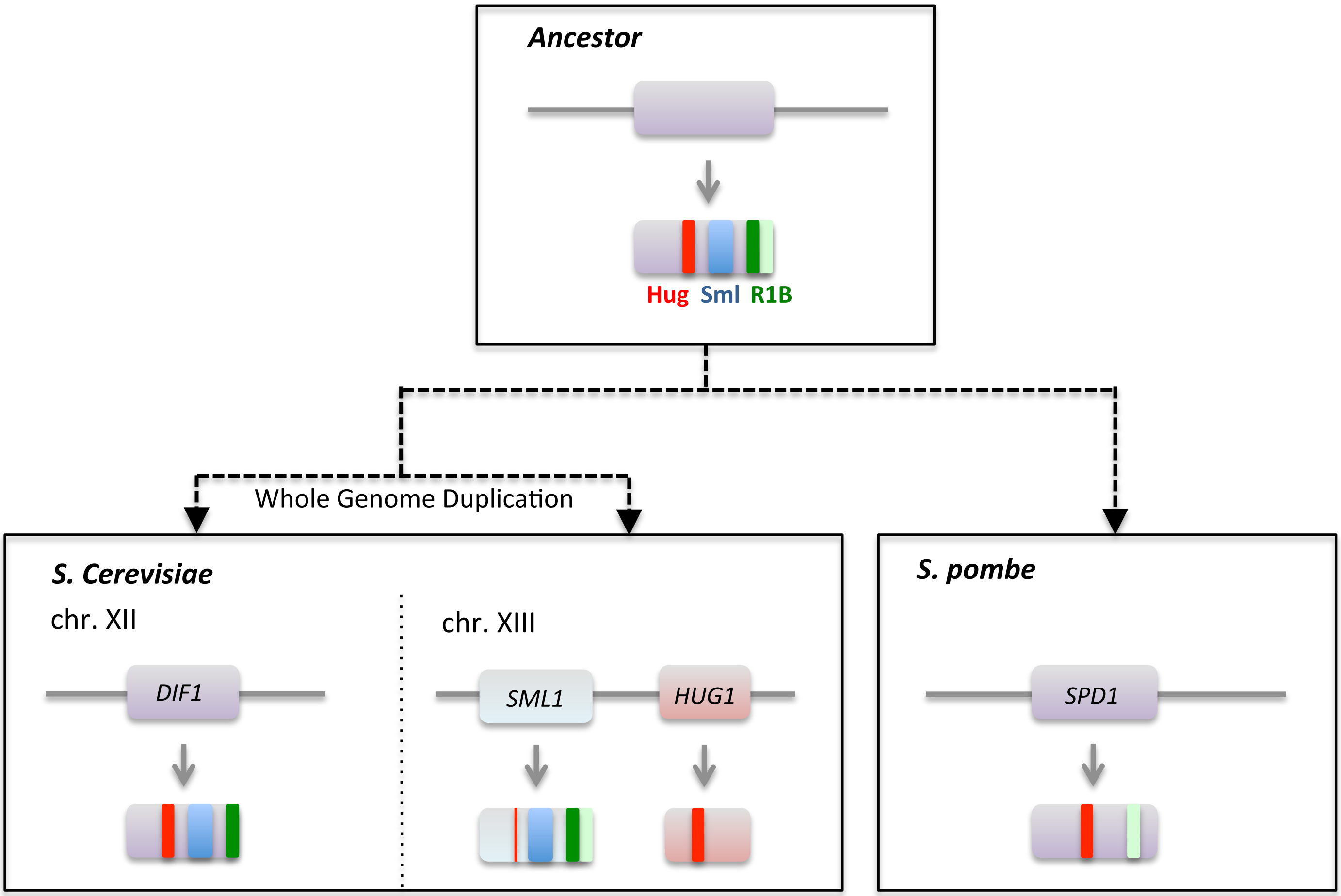

Figure S1: Conservation of RNR regulators in yeast.
Diagrams showing the family of RNR regulators Spd1, Dif1, Sml1 and Hug1 in *S. cerevisiae* and *S. pombe*, and their presumed ancestor. Genes and corresponding proteins are represented as color boxes. The putative ancestor protein contains a Hug domain (Hug, red), an Sml domain (Sml, blue) and an Rnr1-binding (R1B) domain which has been divided into an N-terminal (dark green) and a C-terminal (light green) subdomains.

A.

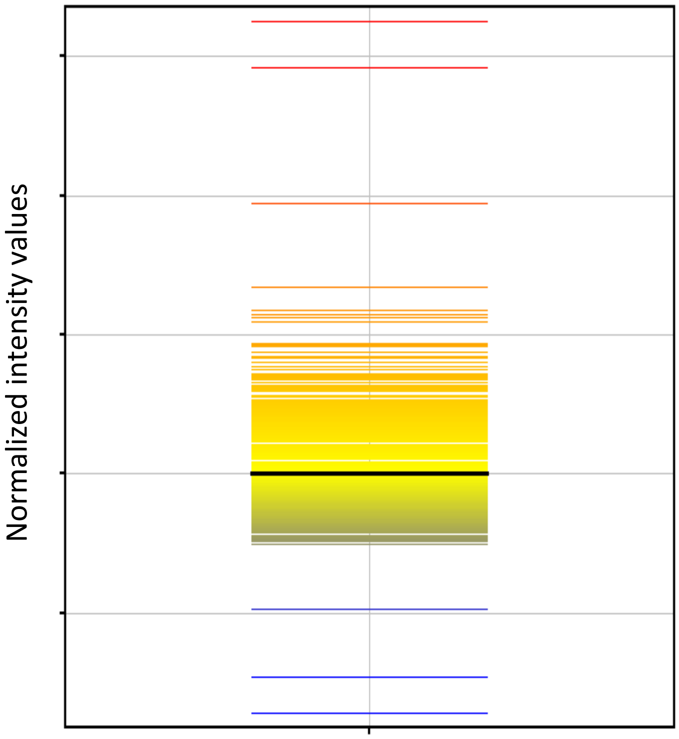

B.

Name	Symbol	adjusted p-value	Fold change	Up/down
YPL153C	*RAD53*	2.30E-03	9.55	+
YML058W-A	*HUG1*	3.77E-05	7.55	+
YDR007W	*TRP1*	1.60E-05	3.84	+
YDR374C	*YDR374C*	2.30E-03	2.53	+
YJR078W	*BNA2*	3.45E-03	2.25	+
YGR243W	*FMP43*	2.30E-03	2.22	+
YPR015C	*YPR015C*	1.83E-04	2.18	+
YIL066C	*RNR3*	7.95E-05	2.14	+
YGR043C	*NQM1*	5.94E-03	1.91	+
YLR327C	*TMA10*	4.39E-03	1.90	+
YNR034W-A	*YNR034W-A*	5.85E-03	1.90	+
YNL194C	*YNL194C*	2.30E-03	1.89	+
YFL014W	*HSP12*	3.62E-03	1.84	+
YHL048W	*COS8*	4.64E-03	3.28	-
YIR043C	*YIR043C*	2.29E-04	2.76	-
YDL248W	*COS7*	3.45E-03	1.96	-

Figure S2 : **Genes which are differentially expressed in the *RAD53-DL* strain.**
A. Boxplot representing normalized intensity values for gene expression in the *RAD53-DL* strain compared with the wild-type strain. Each gene is represented by one line. Significantly down-regulated or up-regulated genes are indicated in blue and red respectively.
B. List of genes, which were significantly up-regulated (expression +) or down-regulated (expression -) in *RAD53-DL* strain. log2 expression ratios (LOWESS normalization) of differentially expressed target genes (Fold change > 1.8, p-value < 0.01) from eight independent experiments are indicated. T-statistics and *P*-values were calculated based on Students' *t*-test.

A.

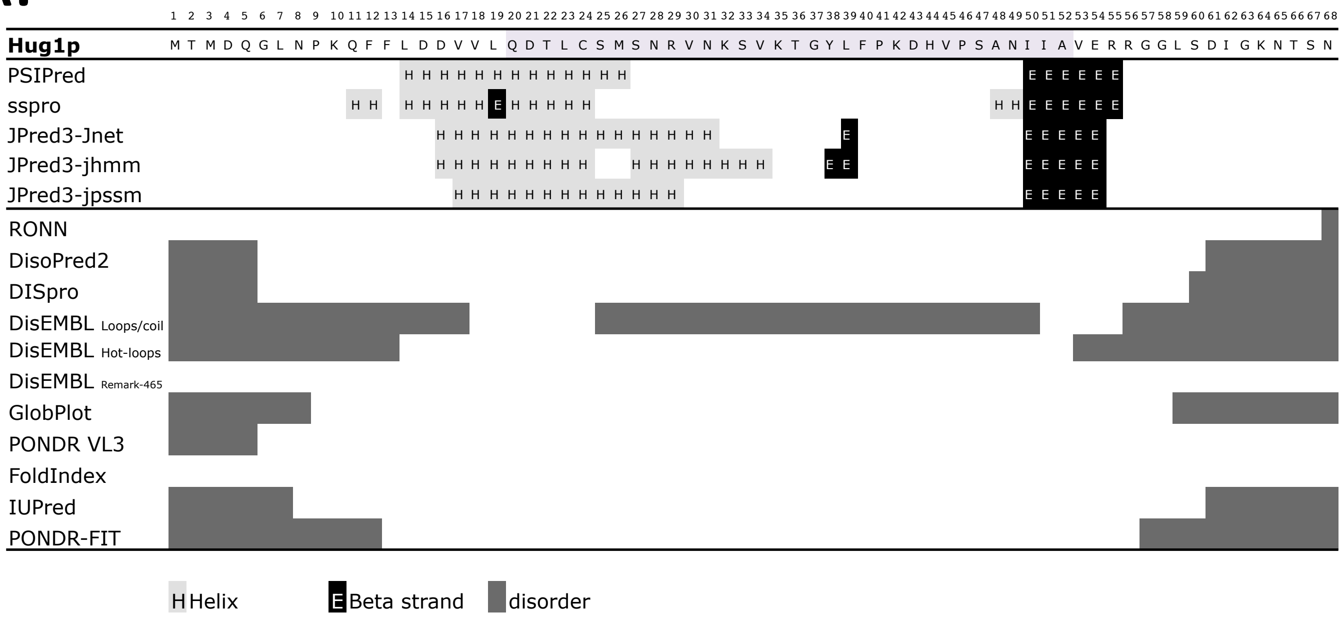

Figure S3 : **Disorder predictions of the Hug1 protein.**
The amino acid sequence of Hug1 is displayed with the Hug domain highlighted in light pink. Its predicted secondary structure and the location of predicted disordered regions are displayed below the sequence. Helical regions and β-strands are displayed with an « H » and a « E » respectively. Predicted disorder is schematically represented by grey lines along the Hug1 sequence. The predicted secondary structure of Hug1 was obtained with PSIpred, Sspro and JPred3 programs. Nine programs were used to predict the disorder tendency of Hug1. See Suppl. Material and Methods for details.

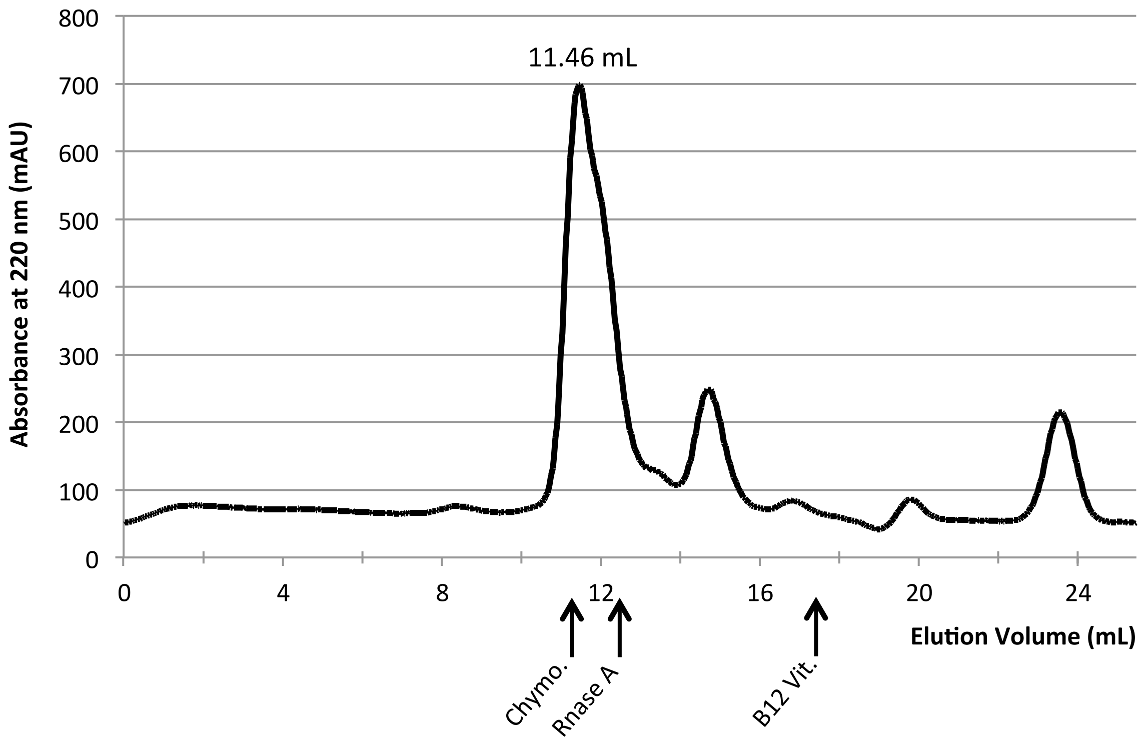

Figure S4: Gel-filtration sizing of recombinant Hug1

Size-exclusion chromatography by fast liquid chromatography (Superdex 75 HR10/300 – GE Healthcare) reveals that 7.5 kDa Hug1 has a larger hydrodynamic radius than 13.7 kDa Ribonuclease A. At the bottom of the chromatogram, we indicated with arrows the reported elution volume for chymotrypsin (Chymo.; 25 kDa), ribonuclease A (Rnase A; 13.7 kDa) and B12 vitamin (B12 Vit.; 1.355 kDa).

A.

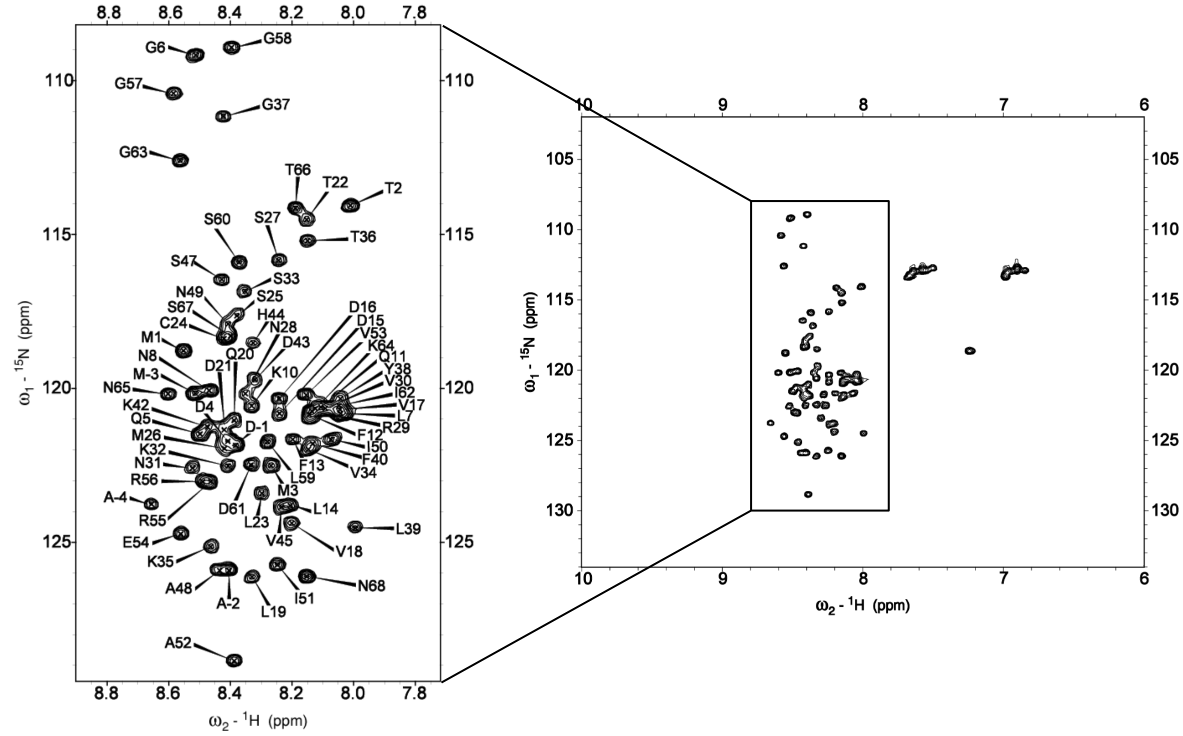
ω2 - 1H (ppm)
ω1 - 15N (ppm)
G6
G58
G57
G37
G63
T66
T22
T2
S60
S27
T36
S47
S33
N49
S25
S67
H44
C24
N28
M1
D43
D16
D15
V53
K64
Q11
N8
D21
Q20
K10
M-3
Y38
V30
I62
V17
N65
K42
D4
Q5
M26
D-1
L7
R29
F12
I50
K32
F13
F40
N31
L59
V34
R56
M3
A-4
D61
L14
R55
L23
L39
E54
V45
V18
K35
A48
A-2
L19
I51
N68
A52

B.

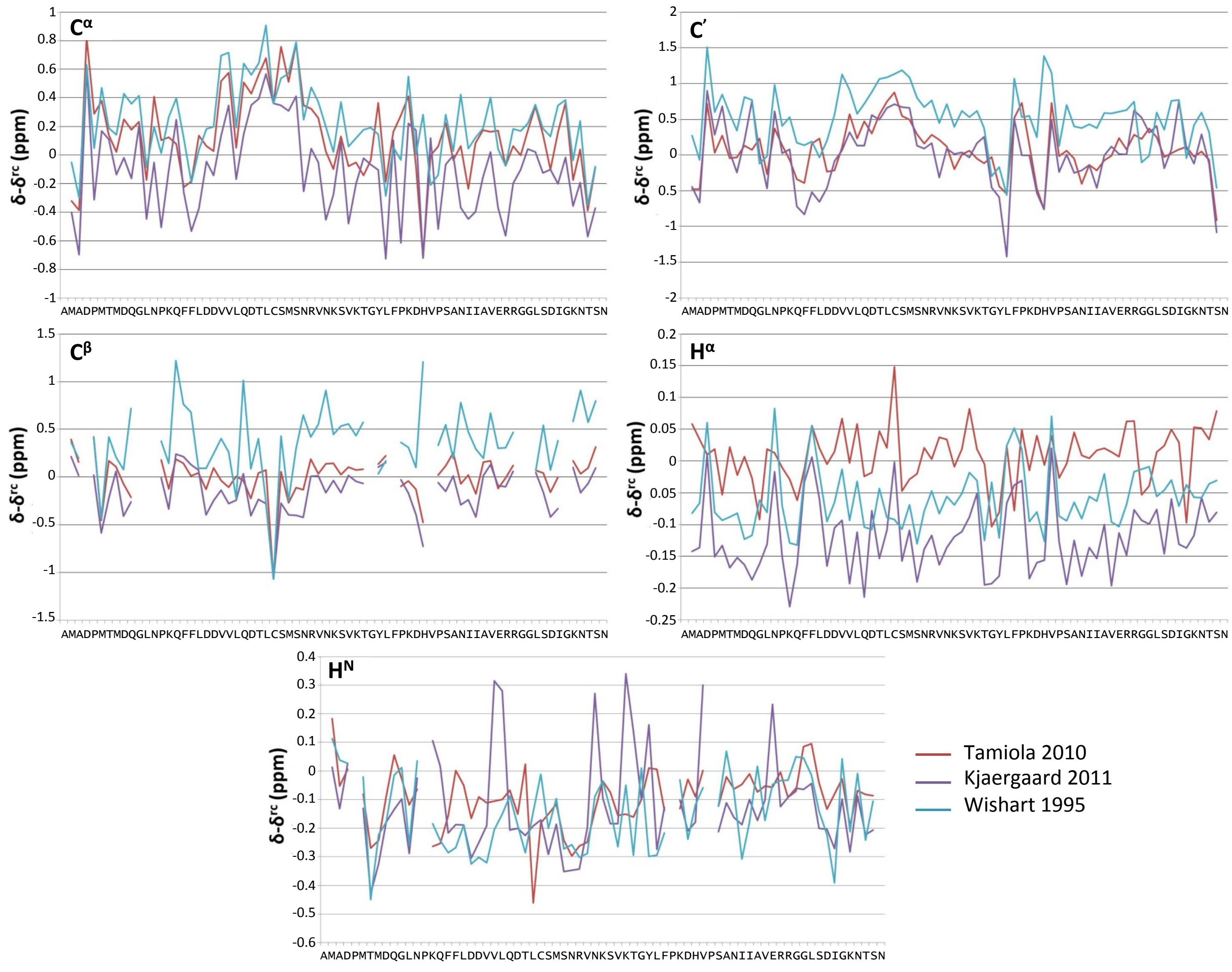
Cα
C′
Cβ
Hα
HN
δ-δrc (ppm)
AMADPMTMDQGLNPKQFFLDDVVLQDTLCSMSNRVNKSVKTGYLFPKDHVPSANIIAVERRGGLSDIGKNTSN
Tamiola 2010
Kjaergaard 2011
Wishart 1995

Figure S5: NMR data indicate that Hug1 is an intrinsically disordered protein but contains a nascent helix spanning V17-S27.

A. 2D ^{15}N, ^{1}H –HSQC of recombinant Hug1 at pH 5.0, 15°C. The lack of chemical shift dispersion is characteristic of unfolded proteins. Assignments indicated on the zoom inset by the one-letter amino acid code followed by the corresponding sequence number along the protein primary sequence were determined using a standard 3D heteronuclear ^{13}C-^{15}N strategy.

B. Secondary chemical shifts relative to random coil of $^{13}C^{\alpha}$ (upper left), $^{13}C'$ (upper right), $^{13}C^{\beta}$ (middle left), $^{1}H^{\alpha}$ (middle right) and $^{1}H^{N}$ (lower center) resonances of Hug1 were calculated by using indicated random coil shift sets (29, 30, 32).

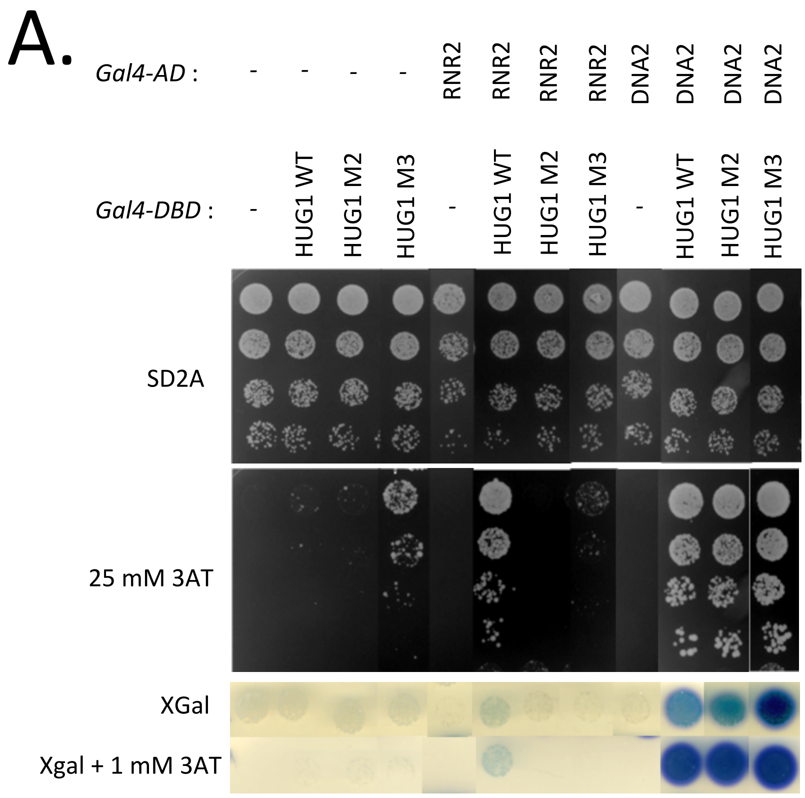

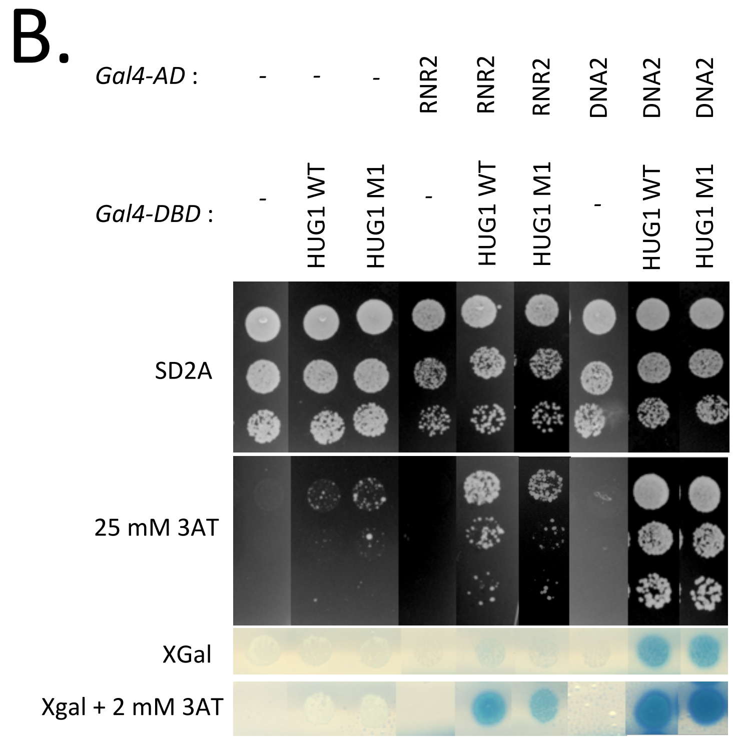

Figure S6: Characterization of the interaction between Hug1 and Rnr2 or Dna2 by yeast two-hybrid assays

Hug1 wt, M1, M2 or M3 mutant was fused to Gal4 DNA-binding domain (Gal4-DBD) and Rnr2 or Dna2 to Gal4 activating domain (Gal4-AD). Empty vectors (-) (pGBT9 and pACT2 respectively) were used as negative controls. Serial dilutions of diploids containing the various combinations of Gal4 fusions were plated in the absence (SD2A) or in the presence of indicated concentration of 3-Amino-Triazol (3AT) to evaluate transcriptional activation of *HIS3*. Blue color formation in the presence of X-gal indicating transcriptional activation of the second reporter gene LacZ was then monitored.

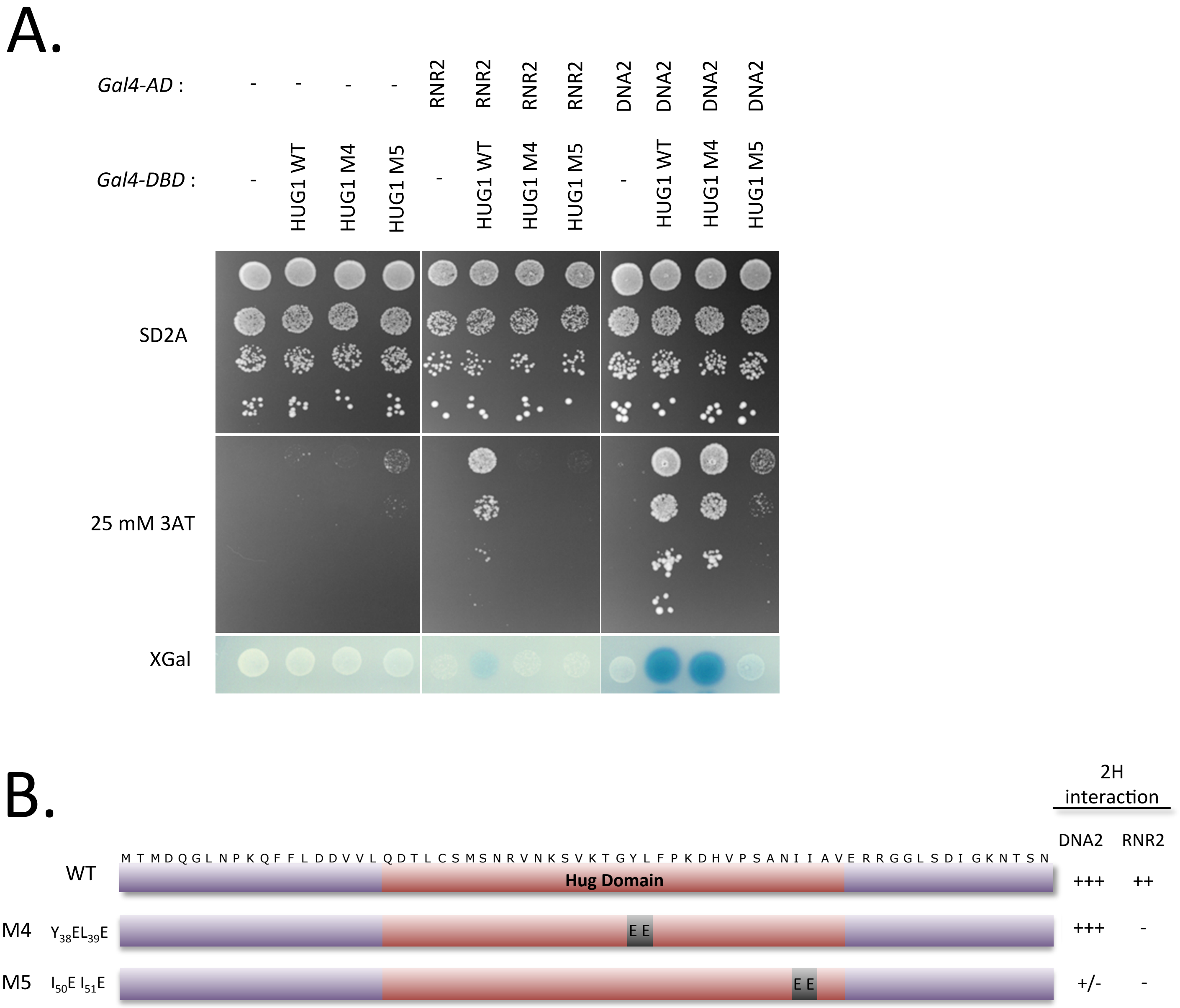

Figure S7: Yeast two-hybrid study of the interaction between Hug1 mutants on residues identified by NMR and Rnr2 or Dna2.

Hug1 wt, M4 or M5 mutant was fused to Gal4 DNA-binding domain (Gal4-DBD) and Rnr2 or Dna2 to Gal4 activating domain (Gal4-AD). Empty vectors (-) (pGBT9 and pACT2 respectively) were used as negative controls. Serial dilutions of diploids containing the various combinations of Gal4 fusions were plated in the absence (SD2A) or in the presence of indicated concentration of 3-Amino-Triazol (3AT) to evaluate transcriptional activation of HIS3. Blue color formation in presence of X-gal indicating transcriptional activation of the second reporter gene LacZ was then monitored. Activation of reporter genes was very high (+++), high (++), very low (+/-) or undetectable (-). The Hug domain is indicated in red.

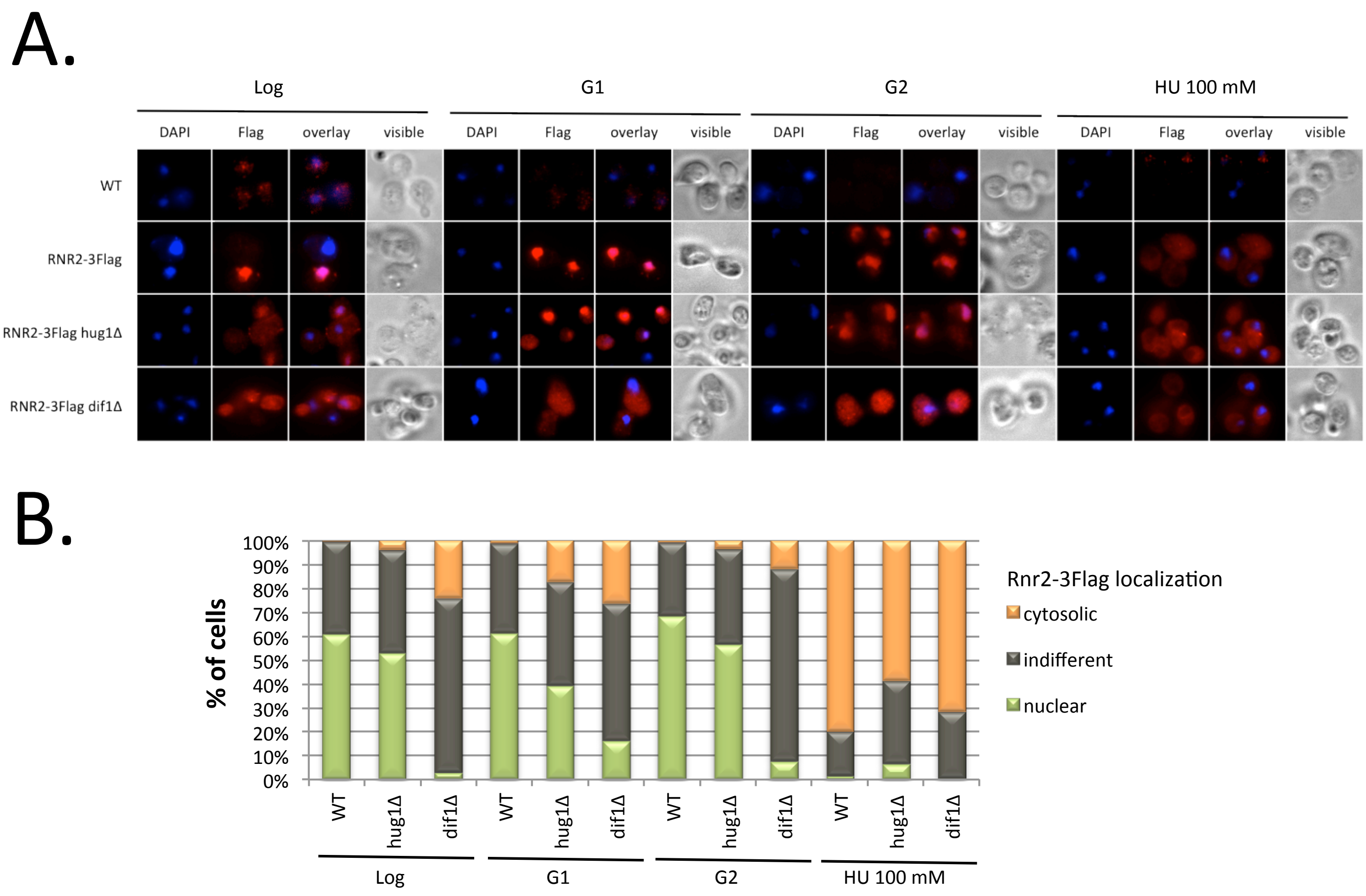

Figure S8: By contrast to Dif1, Hug1 is not required for the nuclear localization of Rnr small subunits.

A. Wild-type (WT), *RNR2-3FLAG*, *RNR2-3FLAG hug1Δ*, *RNR2-3FLAG dif1Δ* cells were grown to log phase (Log) or arrested in G1 with alpha-factor (G1) or arrested in G2 with nocodazole (G2) or treated with 100 mM HU. Fixed cells were then processed for Rnr2 visualization by indirect immunofluorescence. Visualization of nuclei was performed by DAPI staining.

B. Quantitative analysis of the experiment described in A. corresponding to the observation of 200 cells.

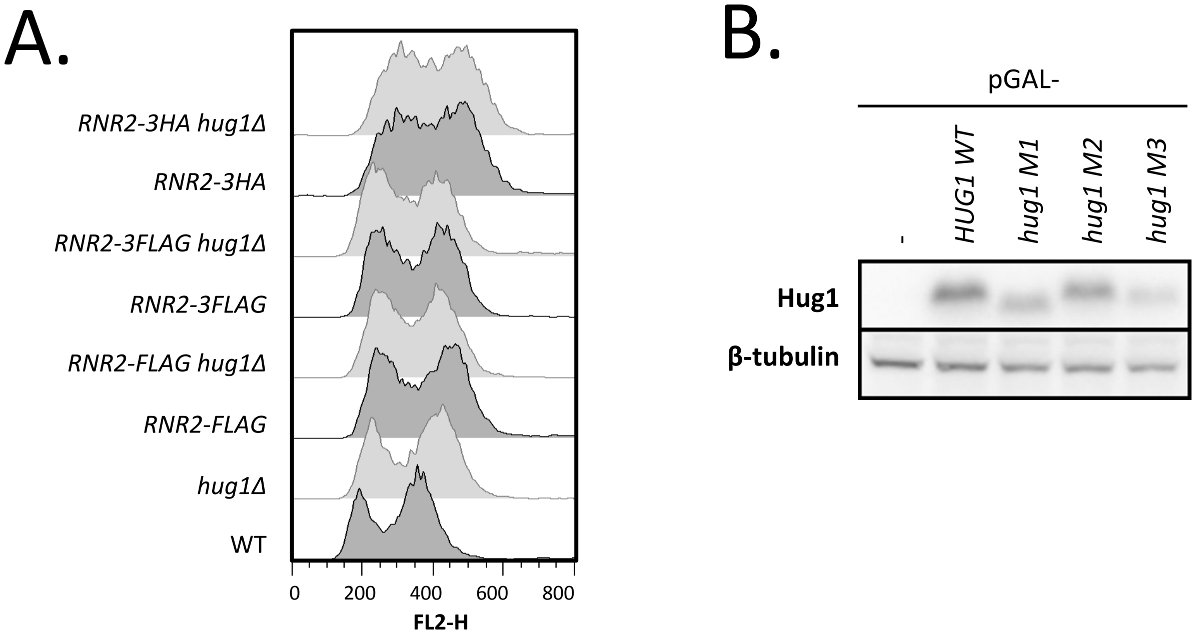

Figure S9: Effect of Hug1 expression on the growth of epitope-tagged Rnr2 strains.
A. Invalidation of *HUG1* does not modify the cell-cycle profiles of exponentially growing epitope-tagged Rnr2 strains. The wild-type (WT) or the indicated epitope-tagged *RNR2* strains, invalidated or not for *HUG1,* were fixed with ethanol while exponentially growing and cell-cycle profiles were analyzed by flow cytometry after propidium iodide staining. FL2-H: Fluorescence intensity of propidium iodide.
B. Hug1 is expressed from pGAL-HUG1 WT and mutant vectors. The *RNR2-3FLAG hug1Δ* strain was transformed with pGAL-HUG1 WT, mutant (M1, M2 or M3) or corresponding empty vector (-). Transformants were grown in minimal medium containing 2% galactose and total protein extracts were prepared from exponentially growing cells. Equal amounts of total protein extracts were loaded and analyzed by SDS-PAGE followed by western blotting using polyclonal anti-Hug1 serum and polyclonal anti-β-tubulin antibodies.

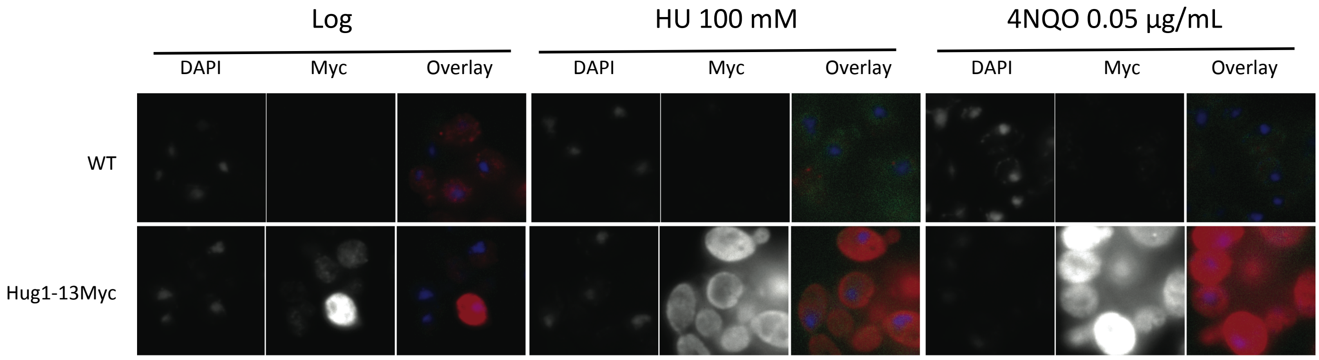

Figure S10: Hug1 is mainly localized in the cytoplasm.
Wild-type (WT) or Hug1-13myc cells were grown to log phase (Log), and treated with 100 mM HU or 4NQO (0.05 µg/mL). Cells were then processed for Hug1 visualization by indirect immunofluorescence. Nuclei were stained with DAPI.

A.

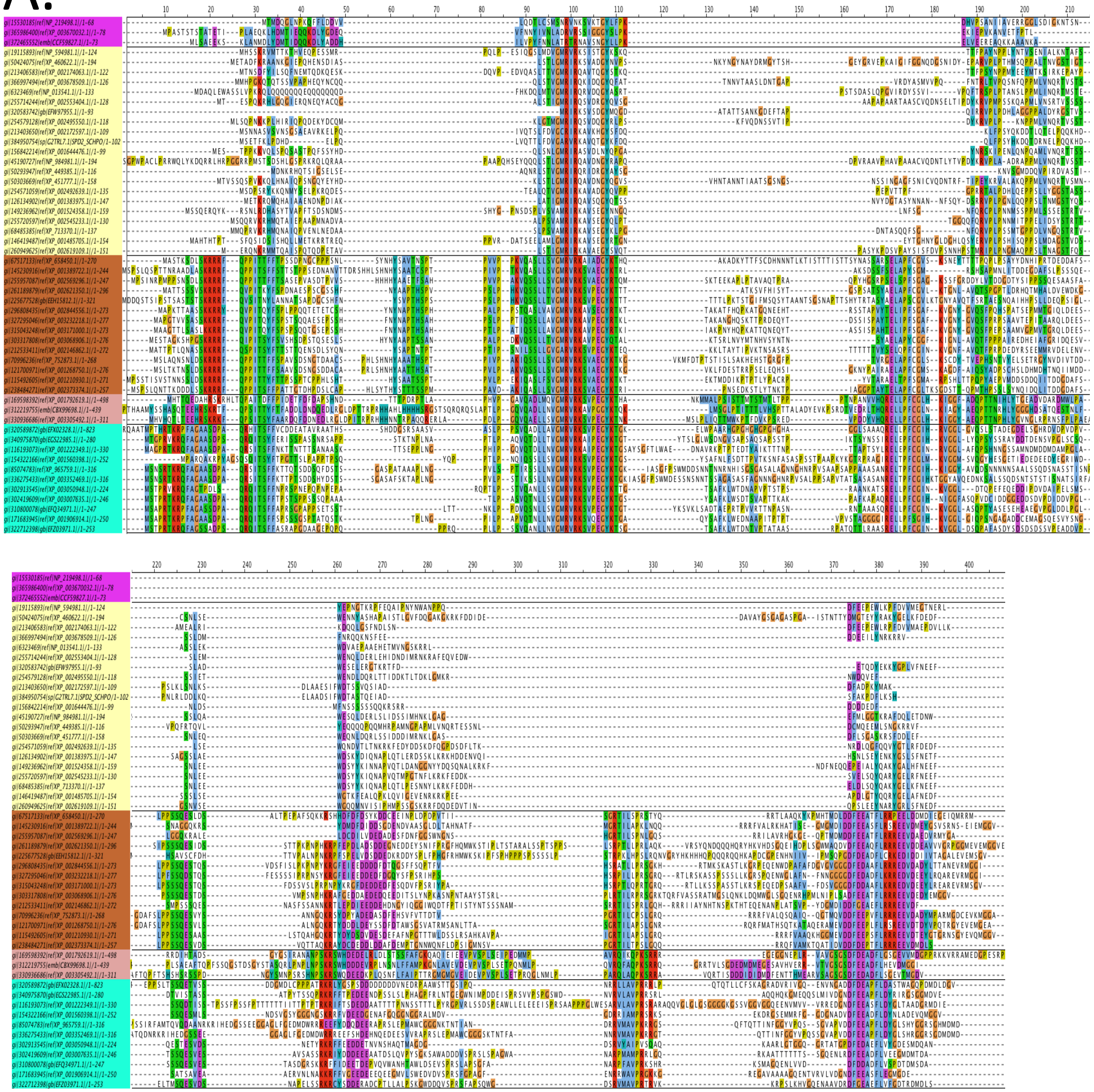

B.

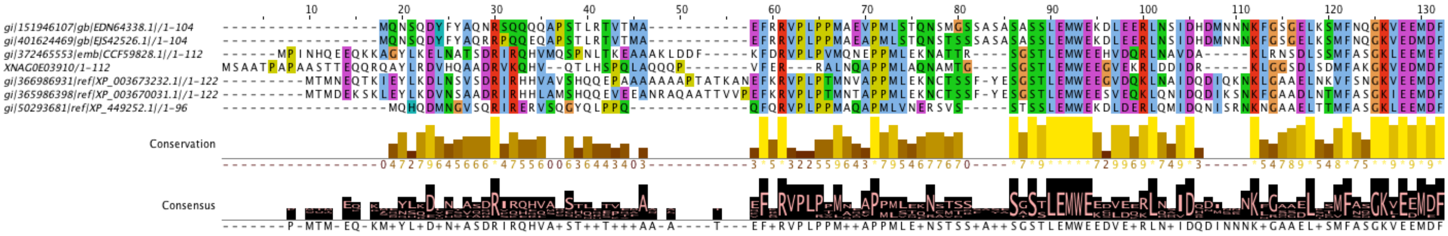

Figure S11: Alignment of Aer122c/ Spd1/Dif1/Sml1/Hug1 proteins.

A. Multiple sequence alignments of the full length sequences were generated using the MUSCLE tool (*http://www.ebi.ac.uk/Tools/msa/muscle//)* and further refined manually. Hug1 family proteins are highlighted in purple.

B. Sequence alignment of Sml1 proteins. Alignments were generated using MUSCLE tool. Consensus sequence is indicated below alignments.

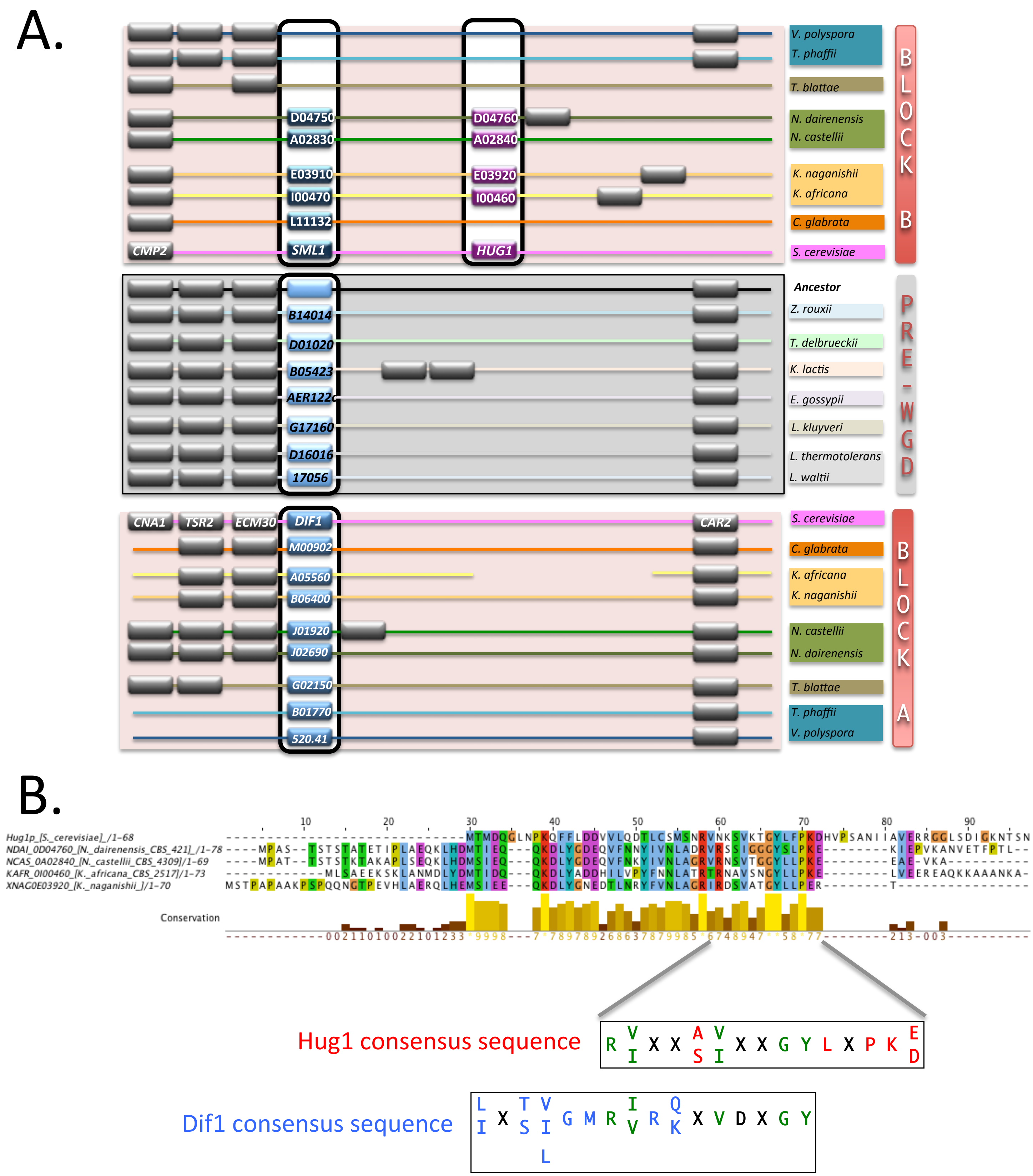
A.
CMP2
SML1
HUG1
D04750
A02830
E03910
I00470
L11132
D04760
A02840
E03920
I00460
V. polyspora
T. phaffii
T. blattae
N. dairenensis
N. castellii
K. naganishii
K. africana
C. glabrata
S. cerevisiae
BLOCK B
B14014
D01020
B05423
AER122c
G17160
D16016
17056
Ancestor
Z. rouxii
T. delbrueckii
K. lactis
E. gossypii
L. kluyveri
L. thermotolerans
L. waltii
PRE-WGD
CNA1
TSR2
ECM30
DIF1
CAR2
M00902
A05560
B06400
J01920
J02690
G02150
B01770
520.41
S. cerevisiae
C. glabrata
K. africana
K. naganishii
N. castellii
N. dairenensis
T. blattae
T. phaffii
V. polyspora
BLOCK A
B.
Hug1p_[S._cerevisiae]_/1-68
NDAI_0D04760_[N._dairenensis_CBS_421]_/1-78
NCAS_0A02840_[N._castellii_CBS_4309]/1-69
KAFR_0I00460_[K._africana_CBS_2517]/1-73
XNAG0E03920_[K._naganishii_]/1-70
Conservation
Hug1 consensus sequence
Dif1 consensus sequence

Figure S12 : *HUG1* gene family.

A. Yeast gene order focused on the *A. gossypii* (or *E. gossypii*) gene *AER122c*. Lines represent chromosomes. Each box represents a gene. Homolog genes are vertically aligned. Genes in focus are colored in blue or purple. Pre-WGD species are represented in the middle panel. Close species are indicated within a unique color box on the right. Blocks A and B represent different chromosomes from indicated post-WGD species. Block A gathers chromosomes containing *DIF1* homologs. Block B gathers together chromosomes containing *SML1* and *HUG1* homologs. Gathering was done based on both synteny and protein sequence similarity. For clarity, every *S. cerevisiae* gene was annotated whereas for other species, only *AER122C*/*DIF1*/*HUG1*/*SML1* family genes were annotated. Yeast Gene Order Browser (http://wolfe.gen.tcd.ie/ygob/) was used for synteny. WGD: Whole Genome Duplication.

B. Alignment of Hug1 proteins. Multiple sequence alignments of the full length sequences corresponding to purple boxes in A were generated using the MAFFT tool (*mafft.cbrc.jp/alignment/)* and further refined manually. A specific part of the Hug domain is highlighted (Hug1 consensus sequence) and compared with the conserved sequence motif in the Dif1 family (Dif1 consensus sequence). Amino acids shared by Dif1 and Hug1 are indicated in green. Residues specific for the Hug1 and Dif1 families are depicted in red and blue respectively. One-letter amino acid code was used. X stands for variable residue.

SUPPLEMENTAL MATERIAL AND METHODS

Transcriptomic Microarray Experiments and Analysis

Total RNA was extracted from RAD53-DL and isogenic wild type log phase cells grown 3 hours in YPD using Phenol/chlorophorme protocol (57). 50 µg of total RNA was reverse transcribed using 5 µg of polydT(12-18) (Amersham), 0.4 mM aa-UTP, 50 mM DTT, 400 units of Super Script II (Invitrogen) and 1X of dNTPs mix (50X : 25 mM dATP, 25 mM dCTP, 25 mM dGTP, 10 mM dTTP). Parental RNA was degraded using RNaseH (2 units) (Invitrogen) and 0.5 µg of RNase A (Sigma). Probes were labeled with 0.1 M of Na-Carbonate and NHS-esther Cy3 or Cy5 (Amersham) and purified using QIAquick Purification kit (Qiagen). Probes were quantified using a nanodrop spectrophotometer ND-1000 (Nanodrop) and hybridized to yeast 60-mer oligonucleotide arrays according to the manufacter's instruction (G4140B, Yeast V2, Agilent technologies). Array slides were then scanned at 10 µm resolution using an Axon GenePix 4000B scanner and GenePix Pro6.0 software. Experiments were performed eight times, including dye-swap technical replicates or/and biological replicates.

Microarrays data were analyzed using GeneSpring GX 10 software. A first global normalization was performed according to LOWESS (Locally Weighted Scatter-plot Smoothing) method. Then a t-test was realized ($t = m/(s/\sqrt{n})$, with m mean intensities, s standard deviation, n experiments number). A threshold of 1.8 was fixed for fold change values. An asymptotic analysis was performed to calculate p-value. The p-value was then adjusted according to Benjamini-Hochberg method (58), the minimum threshold was set at 10^{-2}.

Secondary structure and disorder prediction.

Three programs servers were used to predict the secondary structure of Hug1.

PSIPred : http://bioinf.cs.ucl.ac.uk/psipred/ ; SSpro : http://download.igb.uci.edu/sspro4.html ; JPred3 : http://www.compbio.dundee.ac.uk/www-jpred/

Nine programs were used to predict the disorder tendency of Hug1.

RONN : http://www.strubi.ox.ac.uk/RONN ; DISOPRED2 : http://bioinf.cs.ucl.ac.uk/disopred/ ; DISpro : http://scratch.proteomics.ics.uci.edu/ ; DisEMBL : http://dis.embl.de/ ; GlobPlot : http://globplot.embl.de/ ; PONDR-VL3 : http://www.pondr.com/ ; FoldIndex : http://bip.weizmann.ac.il/fldbin/findex ; IUPred : http://iupred.enzim.hu/ ; PONDR-FIT : www.disprot.org/pondr-fit.php.

Indirect Immunofluorescence

Cells were grown at 30°C to an OD_{600} of 0.25, then treated 2 hours with α-factor (1 µg/mL), nocodazole (15 µg/mL), HU (100 mM) or mock buffer. Cells were fixed by addition of formaldehyde (Sigma) to the culture flask to a final concentration of 3.7% v/v. After incubation for 30 minutes at room temperature, cells were washed once with 1mL of KP Sorbitol buffer (15.5 mM K_2HPO_4, 34.5 mM KH_2PO_4, 0.5 mM $MgCl_2$, 1.2 M "Sorbitol") and resuspended in the same buffer. Cell walls were digested using zymolyase 100T (Seikagaku Biobusiness) (4 mg/mL) in a buffer containing 10% β-mercaptoethanol for 30 minutes at 30°C. Spheroplasts were washed successively with PBS-Fish (PBS1X, 0.4% Gelatin from cold water fish skin (Sigma)), twice with PBS-Triton (PBS-Fish, 0.1% triton X-100 (Euromedex)), and once with PBS-Fish. Cells were incubated with the primary antibody for one hour at room temperature, then washed as previously described. Cells were incubated with secondary antibody (Alexa 594 from Invitrogen) for one hour in the dark at room temperature. Cells were washed with PBS-Fish and twice with PBS-Triton and briefly incubated with 0.5 µg/mL of DAPI (Sigma) in the dark at room temperature. Cells were washed once with PBS-Fish and resuspended in PBS1X before observation using epifluorescence microscope system (Leica DMRXA).

SUPPLEMENTAL REFERENCES

57. Collart,M.A. and Oliviero,S. (2001) Preparation of yeast RNA. *Curr Protoc Mol Biol*, **Chapter 13**, Unit13.12.

58. Hochberg,Y. and Benjamini,Y. (1990) More powerful procedures for multiple significance testing. *Stat Med*, **9**, 811–818.
